# MicroRNAs control mRNA fate by compartmentalization based on 3′ UTR length in male germ cells

**DOI:** 10.1186/s13059-017-1243-x

**Published:** 2017-06-15

**Authors:** Ying Zhang, Chong Tang, Tian Yu, Ruirui Zhang, Huili Zheng, Wei Yan

**Affiliations:** 1 Department of Physiology and Cell Biology, University of Nevada, Reno School of Medicine, Center for Molecular Medicine, Room 207B, 1664 North Virginia Street, MS/0575, Reno, NV 89557 USA; 2 Department of Biology, University of Nevada, Reno, 1664 North Virginia Street, MS575, Reno, NV 89557 USA

**Keywords:** Small RNA, miRNA, 3′ UTR length, Delayed translation, mRNA fate, Germ cell, Fertility, Cytoplasmic compartmentalization

## Abstract

**Background:**

Post-transcriptional regulation of gene expression can be achieved through the control of mRNA stability, cytoplasmic compartmentalization, 3′ UTR length and translational efficacy. Spermiogenesis, a process through which haploid male germ cells differentiate into spermatozoa, represents an ideal model for studying post-transcriptional regulation in vivo because it involves a large number of transcripts that are physically sequestered in ribonucleoprotein particles (RNPs) and thus subjected to delayed translation. To explore how small RNAs regulate mRNA fate, we conducted RNA-Seq analyses to determine not only the levels of both mRNAs and small noncoding RNAs, but also their cytoplasmic compartmentalization during spermiogenesis.

**Result:**

Among all small noncoding RNAs studied, miRNAs displayed the most dynamic changes in both abundance and subcytoplasmic localization. mRNAs with shorter 3′ UTRs became increasingly enriched in RNPs from pachytene spermatocytes to round spermatids, and the enrichment of shorter 3′ UTR mRNAs in RNPs coincided with newly synthesized miRNAs that target these mRNAs at sites closer to the stop codon. In contrast, the translocation of longer 3′ UTR mRNAs from RNPs to polysomes correlated with the production of new miRNAs that target these mRNAs at sites distal to the stop codon.

**Conclusions:**

miRNAs appear to control cytoplasmic compartmentalization of mRNAs based on 3′ UTR length. Our data suggest that transcripts with longer 3′ UTRs tend to contain distal miRNA binding sites and are thus targeted to polysomes for translation followed by degradation. In contrast, those with shorter 3′ UTRs only possess proximal miRNA binding sites, which, therefore, are targeted into RNPs for enrichment and delayed translation.

**Electronic supplementary material:**

The online version of this article (doi:10.1186/s13059-017-1243-x) contains supplementary material, which is available to authorized users.

## Background

Once synthesized, transcripts are subjected to extensive post-transcriptional regulation at both the nuclear and cytoplasmic levels, including processing of the precursor mRNAs (e.g., alternative splicing, alternative polyadenylation, RNA editing, etc.), nucleocytoplasmic transport, sequestration of transcripts in ribonucleoprotein particle (RNP)-enriched cytoplasmic compartments (e.g., p-body, nuage, stress granules, chromatoid body, etc.), so that mRNA stability, subcellular localization and translational efficiency can be coordinated to fulfill specific cellular functions [1–5]. As one of the most common means of mRNA fate control, compartmentalization of mRNAs in the cytoplasm plays a critical role in post-transcriptional regulation of gene expression [3, 6, 7]. mRNAs sequestered into various subcytoplasmic compartments are believed to be further processed and/or modified such that they either get translated or become stabilized and/or translationally suppressed through binding to a large number of RNA-binding proteins (RBPs) at the 3′ UTRs [3, 8–10]. In addition to RBPs, small noncoding RNAs (sncRNAs), e.g., miRNAs, endo-siRNAs, and piRNAs, are also involved in the mRNA fate control, which is not surprising given that sncRNAs, like RBPs, also bind to 3′ UTRs of their target mRNAs and affect mRNA stability and translational activity [11–14].

Another emerging mechanism of post-transcriptional regulation of gene expression lies in the control of 3′ UTR length. For example, recent studies have discovered that the nonsense-mediated mRNA decay (NMD) pathway, known to serve as a quality control mechanism for mRNAs, also functions to regulate the 3′ UTR length during spermiogenesis by eliminating transcript isoforms with longer 3′ UTRs, thus enriching those with shorter 3′ UTRs [15–17]. The impact of 3′ UTR length on the stability and translational efficiency of mRNAs is likely due to the fact that longer 3′ UTRs allow for binding by more RBPs and sncRNAs [18–20]. Therefore, transcripts with longer 3′ UTRs may have a reduced translational efficiency, but their stability would be enhanced, thus allowing for higher orders of regulation of translational timing and amplitude. This may explain why brain neuronal cells tend to have a transcriptome enriched with longer 3′ UTR transcripts compared to other somatic cell types [21, 22]. In contrast, shorter 3′ UTRs bind fewer RBPs and sncRNAs; thus, the translational efficiency could be drastically enhanced to accommodate quick turnover in protein production and function [23]. A global 3′ UTR shortening event has been observed during late meiotic and haploid phases of spermatogenesis, where a large number of transcripts are transcribed without being translated; instead, those mRNAs are sequestered in the RNP-enriched structure called nuage, or intermitochondrial cement, in pachytene spermatocytes (late meiotic male germ cells) and in the chromatoid body (CB) in round spermatids (early haploid male germ cells) for an extended period of time (several days to up to 2 weeks) [19, 24–26]. The physiological significance of such a profound delay in translation lies in the fact that transcription has to cease as soon as nuclear condensation commences in step 9 spermatids, although many proteins are still needed for the remaining seven steps of spermatid differentiation in mouse testes [26–28]. Those proteins have to be translated using the transcripts synthesized and stored in RNPs *prior to* step 9 spermatids. Therefore, the translationally suppressed RNP transcripts are mostly those needed for the final nine steps of sperm assembly (steps 7–16). Disruptions of the delayed translation cause spermiogenic arrest and male infertility [29–31]. Since the cell types and the timing of translational suppression are well defined, spermiogenesis represents an excellent model for studying the mechanism underlying delayed translation in vivo [28, 32].

Relative enrichment of mRNAs in RNPs and polysomes during late meiotic and haploid phases of spermatogenesis has been studied using microarray-based mRNA profiling analyses [33]. However, the study was conducted using total testes at different developmental stages instead of spermatogenic cells purified from adult testes. Therefore, the transcriptomic data represent gene expression profiles of both testicular somatic (Sertoli, Leydig, and peritubular myoid cells) and germ/spermatogenic (spermatogonia, spermatocytes, and spermatids) cell types, thus complicating the data interpretation. Moreover, the microarray data do not allow for bioinformatic analyses of mRNA structural features, e.g., the lengths of 5′ UTRs, coding sequences, and 3′ UTRs, and provide no information on the expression levels of individual isoforms for genes with multiple transcripts. Although sncRNAs are known to act mainly at post-transcriptional levels, the relationship between sncRNAs and mRNAs subjected to translational delay has not been investigated. More importantly, it remains an outstanding question how 3′ UTR length control fits into the overall theme of cytoplasmic compartmentalization as a critical post-transcriptional regulatory mechanism during spermiogenesis (i.e., the process through which spermatids differentiate into spermatozoa).

To fill these knowledge gaps, we conducted comprehensive transcriptomic profiling analyses on three spermatogenic cell types (pachytene spermatocytes and round and elongating spermatids) purified from adult mouse testes using RNA-Seq, and we determined not only the levels of both mRNAs and sncRNAs, but also their cytoplasmic compartmentalization. Bioinformatics analyses revealed miRNAs were mostly enriched in RNPs, and RNP-enriched miRNAs preferentially target RNP-enriched mRNAs. More interestingly, we found that miRNAs could distinguish shorter and longer 3′ UTR transcripts based on the distance between their binding sites and the stop codon. Overall, our genome-wide transcriptomic and bioinformatics analyses have uncovered a highly likely mechanism through which miRNAs shape the haploid male germ cell-specific transcriptome characterized by RNP-enrichment of transcripts with shorter 3′ UTRs.

## Results

### Cycloheximide supplementation is essential for the detection of polyribosome-associated RNAs in purified spermatogenic cells

**Fig. 1:**
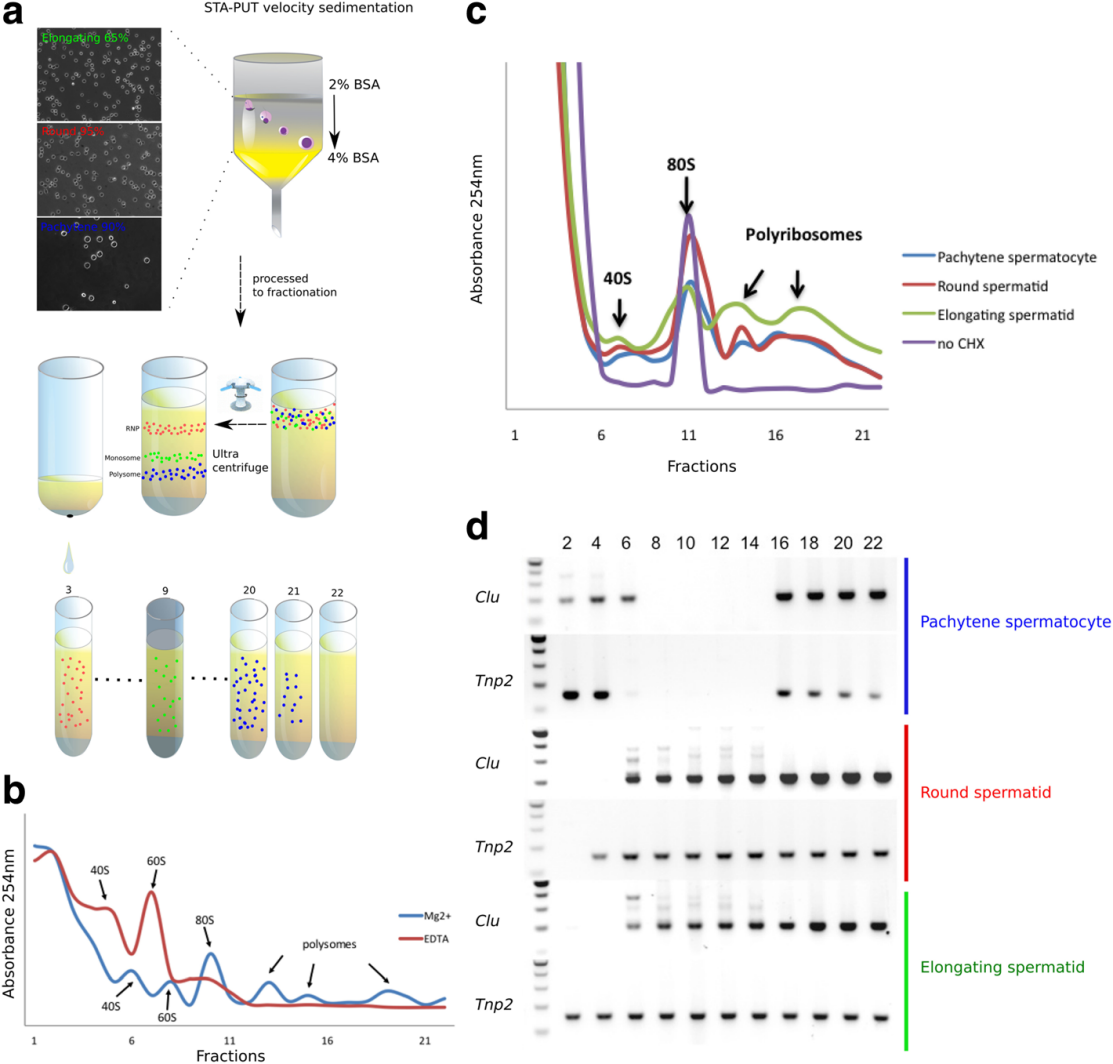
Purification and fractionation of cytoplasmic contents and RNA isolation from pachytene spermatocytes, round spermatids, and elongating spermatids. **a** The STA-PUT method used for purification of pachytene spermatocytes, round spermatids, and elongating spermatids from adult mouse testes, and the sucrose gradient ultracentrifugation-based fractionation procedure for isolating RNAs enriched in ribonuclear particles (RNPs) and polysomes. The morphology and purity of the three types of spermatogenic cells isolated are shown in images at the *top left*. **b** RNA distribution in RNP (fractions 1–4), monosome (fractions 5–15), and polysome (fractions 16–22) fractions of total testis lysates. In the presence of EDTA, RNA and polysomes disassociate, leading to the disappearance of polysome peaks and an increase of monosome peaks. We collected the RNP and polysome fractions in this study. **c** RNA distribution in RNP (fractions 1–4), monosome (fractions 5–15), and polysome (fractions 16–22) fractions of purified pachytene spermatocytes and round and elongating spermatids. Please note that, in the absence of cycloheximide (*CHX*), in the buffer during cell purification, the polysome peaks disappeared. **d** Distribution of *Tnp2* (a male germ cell-specific gene known to display delayed translation during spermiogenesis) and *Clu* (a ubiquitous gene known to be translated throughout spermatogenesis) among all the fractions collected from three spermatogenic cell types

To perform RNA-Seq analyses, we purified pachytene spermatocytes and round and elongating spermatids from wild-type adult testes using the STA-PUT method [34] (Fig. 1a). Based on cell morphology, the purity for pachytene spermatocytes and round and elongating spermatids was estimated at 90, 95, and 65%, respectively (Fig. 1a). Using a sucrose gradient centrifugation protocol [33, 35], we fractionated the cytoplasmic contents into 22 fractions, from which large and small RNAs associated with RNPs (fractions 1–4) and polysomes (fractions 16–22) were isolated for RNA-Seq analyses. By measuring OD_254_, three peaks, representing RNP, mono-ribosome, and poly-ribosome fractions, were observed (Fig. 1b). When the fractionation buffer was supplemented with EDTA, both transcripts and polysomes became disassociated, leading to the disappearance of RNA peaks in the polysome fractions, demonstrating that the polysome-associated RNAs under the EDTA-free conditions are truly the ones specifically bound to polysomes.

We then fractionated the purified spermatogenic cells followed by RNA isolation. However, we noticed that RNAs in the polysome fractions of the purified spermatogenic cells were largely non-existent (Fig. 1c); this has been suggested previously to result from cessation of transcription, but continuous translation when cells are processed in vitro for an extended period, because the mRNAs already loaded onto the polyribosomes can be exhausted rather quickly [36, 37]. To block translation, we supplemented the cell purification and fractionation buffers with cycloheximide (CHX) and, interestingly, the polysome peaks reappeared after fractionation using the purified spermatogenic cells (Fig. 1c). The distribution of *Clu* (a gene known not to be subjected to delayed translation) and *Tnp2* (a gene known to be translationally delayed) [33] further demonstrated the proper fractionation of the cytoplasmic contents into RNP (i.e., translationally suppressed) and polyribosome (i.e., translationally active) fractions using purified spermatogenic cells.

### miRNAs are mostly enriched in RNPs from pachytene spermatocytes to round spermatids and the shift of miRNAs from RNPs to polysomes coincides with active translation in elongating spermatids

**Fig. 2:**
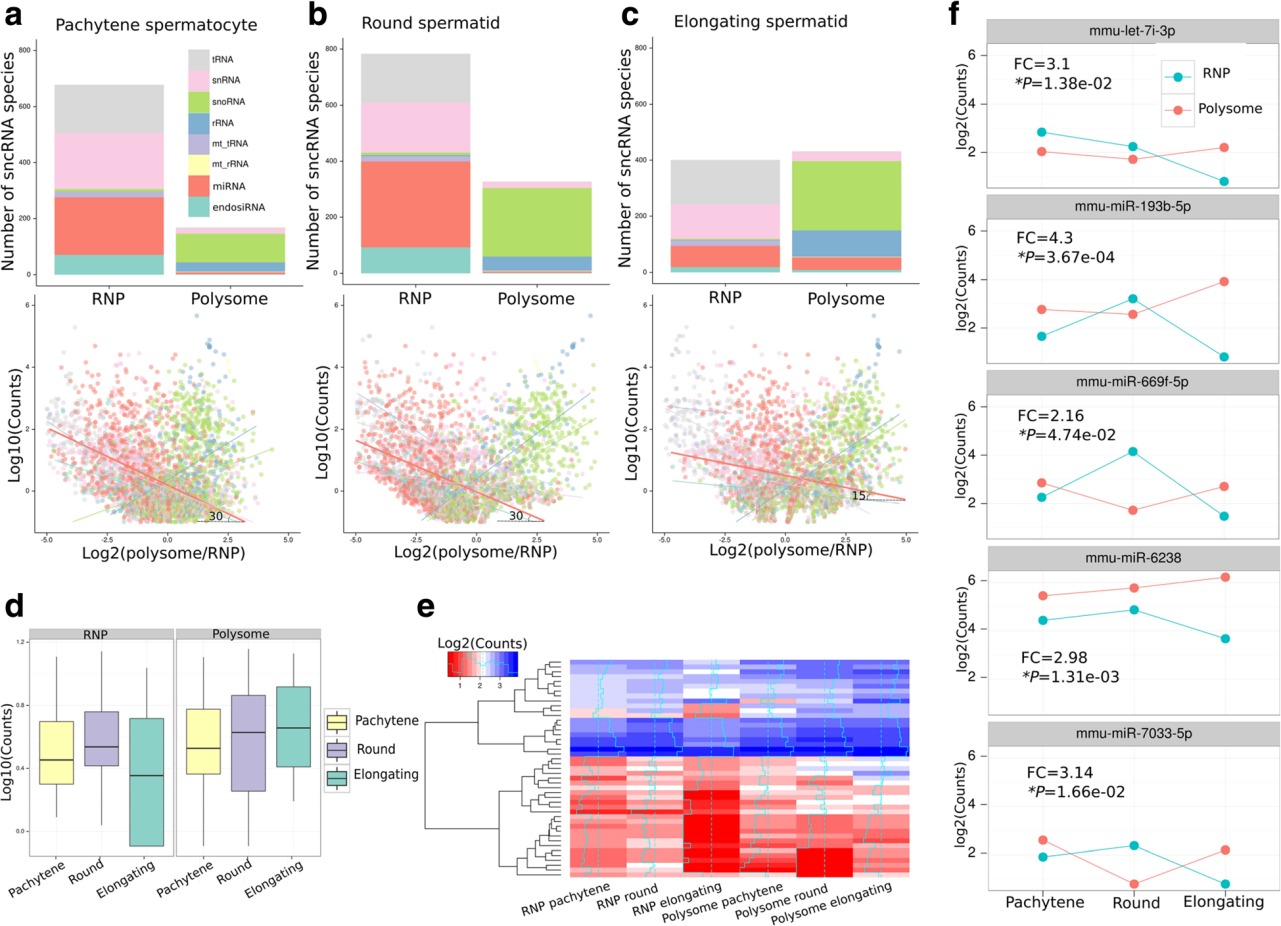
Distribution of small noncoding RNAs (*sncRNAs*) in RNP and polysome fractions in pachytene spermatocytes, round spermatids, and elongating spermatids. **a**–**c** sncRNAs preferentially enriched in RNP or polysome fractions in pachytene spermatocytes (**a**), round spermatids (**b**), and elongating spermatids (**c**). *Upper panels*: RNP-enriched sncRNAs were defined by log_2_(Levels in polysome/Levels in RNP) <0 (Student’s *t*-test, *p* < 0.05), whereas polysome-enriched sncRNAs were those with log_2_(Levels in polysome/Levels in RNP) >0 (Student’s *t*-test, *p* < 0.05). The *y-axis* represents the total number of fraction-enriched sncRNA species. *Lower panels*: dot plots showing correlations between actual expression levels and the ratio of polysome levels to RNP levels of sncRNAs. The *y-axis* shows the log_10_ values of all sncRNA expression counts. The *x-axis* represents the log_2_ values of the ratio of polysome levels to RNP levels. The sncRNAs with higher expression levels in the RNP fraction tend to locate to the *left*, whereas sncRNAs with higher expression levels in the polysome fraction are clustered towards the *right*. Linear regression lines for each group are plotted over the data points. Note the angle of the regression lines of miRNAs are ~30° in pachytene spermatocytes (**a**) and round spermatids (**b**), whereas it decreased to ~15 ° in elongating spermatids (**c**), suggesting a shift of miRNAs from RNP to polysome fractions in elongating spermatids. **d** Boxplots showing average levels of the miRNAs shifting from RNP to polysome fractions in elongating spermatids. The average counts (*y-axis*) of those miRNAs increased in the polysome fraction but decreased in the RNP fraction from round to elongating spermatids. **e** Heat map showing levels of the miRNAs shifting from RNP to polysome fractions in elongating spermatids. Values of log_2_ (RPKM) are presented as variable colors from *red* to *blue*. These miRNAs were upregulated in the polysome fraction and simultaneously downregulated in the RNP fraction from round to elongating spermatids. **f** Expression profiles of the five miRNAs displaying a shift from RNP to polysome in elongating spermatids. All five miRNAs were upregulated in the polysome fraction but downregulated in the RNP fraction from round to elongating spermatids. Student’s *t*-test was used to evaluate statistical significance, and *p* values and fold changes (*FC*) are marked. *Asterisks* indicate statistically significant *p* values

Using small RNAs isolated from the RNP and polyribosome fractions of purified pachytene spermatocytes, round spermatids, and elongating spermatids, we conducted RNA-Seq analyses to profile small RNAs. We annotated eight known small RNA species, including tRNA-derived small RNAs (tsRNAs), small nuclear RNAs (snRNAs), small nucleolar RNAs (snoRNAs), ribosome RNA-derived small RNAs (rRNAs), mitochondrial tRNA-derived small RNAs (mt_tRNAs), mitochondrial rRNA-derived small RNAs (mt_rRNAs), microRNAs (miRNAs), and endogenous small interference RNAs (endo-siRNAs). We also determined their expression levels and relative enrichment in RNPs and polysomes (Fig. 2). Among all the small RNAs identified, piRNAs were the most abundant in both RNPs and polysomes (Additional file 1: Figure S1). Since no significant piRNA enrichment was noted in any of the three cell types (Additional file 1: Figure S1), we did not investigate piRNAs in the present study. To demonstrate the movement of other small RNA species between RNP and polysome fractions during spermiogenesis, we first identified the RNP-enriched sncRNAs [defined as log_2_(Polysome counts/RNP counts) <0, counts >1, Student’s *t*-test, *p* < 0.05] and polysome-enriched sncRNAs [log_2_(Polysome counts/RNP counts) >0, counts >1, Student’s *t*-test, *p* < 0.05]. In both pachytene spermatocytes and round spermatids, snRNAs, tsRNAs, miRNAs, and endo-siRNAs were predominantly enriched in RNPs (Fig. 2a, b). Conversely, rRNAs and snoRNAs were mainly accumulated in the polysomes in these two cell types, consistent with their roles in translation [38, 39] (Fig. 2a, b). When round spermatids develop into elongating spermatids, a significant proportion (~37%) of miRNAs, in addition to snoRNAs and rsRNAs, became enriched in polysomes, and this polysome enrichment of miRNAs was not observed in either spermatocytes or round spermatids (Fig. 2c). By analyzing their levels based on the sncRNA-Seq data, we found that these miRNAs were highly expressed in both RNPs and polysomes in pachytene spermatocytes and round spermatids, but drastically shifted to polysomes in elongating spermatids (Fig. 2d, e). By analyzing the expression levels of five such miRNAs in RNPs and polysomes of the three spermatogenic cell types, we found that their levels decreased in RNPs and increased in polysomes from round to elongated spermatids (Fig. 2f), further supporting that these miRNAs indeed represent those shifting from RNPs to polysomes in elongating spermatids. However, it is noticeable that many other miRNAs remained enriched in RNPs in elongated spermatids (Fig. 2c). Taken together, our small RNA profiling data demonstrate the following: 1) an increasing number of miRNAs became enriched in RNPs from pachytene spermatocytes to round spermatids, coinciding with the accumulation of haploid transcripts that are subjected to translational suppression in RNPs; 2) a large number of miRNAs shifted from RNPs to polyribosomes in elongating spermatids, coinciding with increased translational activity of haploid transcripts in late spermiogenesis; 3) polysome-enriched miRNAs in elongating spermatids represent those coming out of RNPs because their levels decreased in RNPs and increased in polysomes during late stages of spermiogenesis.

### 3′ UTRs of RNP-enriched mRNAs become increasingly shorter compared to those accumulated in polysomes from pachytene spermatocytes to round and elongating spermatids

Using large RNAs isolated from the RNP and polyribosome fractions of purified pachytene spermatocytes, round spermatids, and elongating spermatids, we conducted RNA-Seq analyses to profile mRNAs. To quantitatively compare expression levels of large and small RNAs in RNP versus polysome fractions, we selected 24 endogenous mRNAs which displayed comparable levels in both RNP and polysome fractions (Additional file 1: Figure S2) as internal control for linear regression normalization of the RNA-Seq data [40]. This normalization method was further validated using the other two normalization methods (geometric and quantile normalization). All three normalization methods produced similar scale factors, and normalization between the same (RNP versus RNP or polysome versus polysome) or different (RNP versus polysome) fractions also resulted in similar results (Additional file 1: Figure S3), demonstrating the validity of our normalization method and the robustness of our data.

By analyzing the distribution of mRNAs in RNP and polysome fractions in late pachytene spermatocytes and round and elongating spermatids, we found that the number of mRNAs that were preferentially enriched in RNPs was almost twice that in polysomes in pachytene spermatocytes (679 mRNAs enriched in RNPs versus 355 in polysomes) and elongating spermatids (793 in RNPs versus 422 in polysomes), whereas similar numbers of mRNAs accumulated in either of the two fractions in round spermatids (762 in RNP versus 752 in polysomes) (Additional file 1: Table S1). By comparing the average length of mRNAs in RNPs and polysomes from the same spermatogenic cell type, we determined that the RNP-enriched transcripts were ~700 nucleotides shorter than those abundant in the polysome fractions in pachytene spermatocytes (Additional file 1: Table S2; Student’s *t*-test, *p* = 4.6e-5, and Wilcoxon rank sum test, *p* = 2.16e-7). However, in round spermatids, the average length of the RNP-enriched mRNAs appeared to be much shorter (by ~1376 nucleotides) than that of the polysome-enriched transcripts (Additional file 1: Table S2; Student’s *t*-test, *p* < 2.2e-16, and Wilcoxon rank sum test, *p* < 2.2e-16). Similarly, the average length of RNP-enriched transcripts was ~400 nucleotides shorter than those polysome-enriched ones in elongating spermatids (Additional file 1: Table S2; Student’s *t*-test, *p* = 2.5e-2, and Wilcoxon rank sum test, *p* = 6.6e-6).

Using SpliceR [15, 41], we extracted 5′ and 3′ UTR sequences for the Cufflink-assembled mRNA transcripts (69,013 mRNAs). To validate and correct the end of 3′ UTRs, we compared the 3′ UTR sequences of the 69,013 transcripts with those in a mouse testis polyadenylation site sequencing (PAS) dataset downloaded from the Gene Expression Omnibus (GEO) database at the NCBI (accession GSM747485). Interestingly, ~90% of our Cufflink-assembled transcripts contained correct 3′ UTR ends. We chose four transcripts (*Cebpg*, *Eif4h*, *Ubp1*, and *Hip1*) to demonstrate this validation procedure (Additional file 1: Figure S4). This “correction” procedure further improved the accuracy of the 3′ UTR length analyses.

**Fig. 3:**
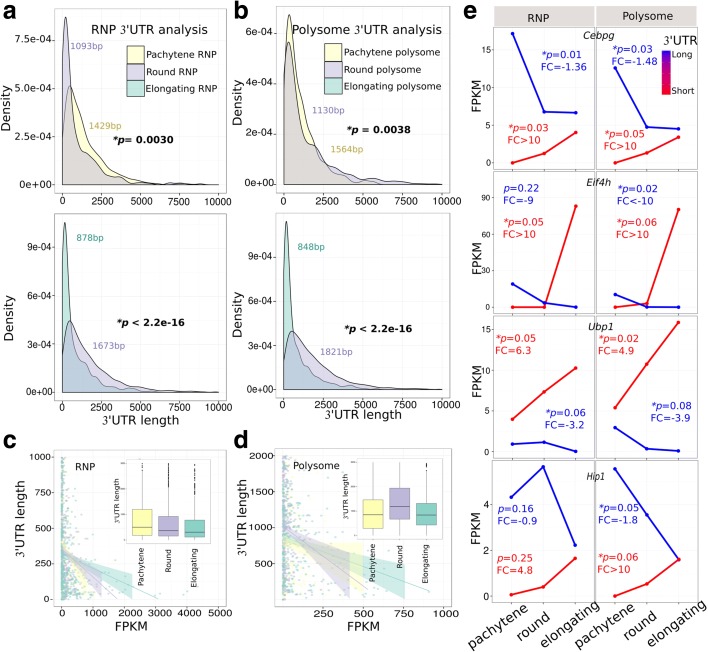
Dynamic 3′ UTR shortening during late meiotic and early haploid phases of spermatogenesis. **a** Density plots showing changes in 3′ UTR length of RNP-enriched mRNAs from pachytene spermatocytes to round (*upper panel*) and from round to elongating spermatids (*lower panel*). Note that the 3′ UTR length was deduced based on the total length and the position of the stop codon of all SpliceR CDS transcripts. The RNP-enriched transcripts were defined as those with upregulated levels in the RNP of one cell type when compared to another (Student’s *t*-test and Wilcoxon rank sum test). The 3′ UTR length of RNP-enriched transcripts becomes increasingly shorter from pachytene spermatocytes to round, and then to elongating spermatids. **b** Density plots showing changes in the 3′ UTR length of polysome-enriched mRNAs from pachytene spermatocytes to round (*upper panel*) and from round to elongating spermatids (*lower panel*). The polysome-enriched transcripts are defined as those with upregulated levels in the polysome fractions of one cell type when compared to another (Student’s *t*-test and Wilcoxon rank sum test). While the 3′ UTR length of polysome-enriched transcripts becomes significantly shorter from round to elongating spermatids, this change does not appear to be significant between pachytene spermatocytes and round spermatids. **c** Correlation between 3′ UTR length and expression level in the RNP fractions of pachytene spermatocytes and round and elongating spermatids. The RNP-enriched transcripts are defined as those significantly upregulated in RNPs compared to polysomes (Student’s *t*-test and Wilcoxon rank sum test) in each of the three cell types. The regression lines were plotted to show the average expression levels of RNP-enriched mRNAs. Note that the transcripts enriched in elongating spermatid RNP fractions display significantly shorter 3′ UTRs but higher expression levels. Boxplots if the inset demonstrate that the 3′ UTR length of RNP-enriched transcripts was increasingly shorter from pachytene spermatocytes to round and elongating spermatids (Student’s *t*-test and Wilcoxon rank sum test, *p* values ranged from 2.2e-16 to 2.3e-3; see Additional file 1: Table S4 for details). **d** Correlation between 3′ UTR length and expression levels in polysome fractions of pachytene spermatocytes and round and elongating spermatids. The polysome-enriched transcripts are defined as those significantly upregulated in polysome compared to RNP fractions (Student’s *t*-test and Wilcoxon rank sum test; see Additional file 1: Table S4 for *p* values) in each of the three cell types. The regression lines were plotted to show the average expression levels of polysome-enriched mRNAs. Note that the transcripts enriched in elongating spermatid polysome fractions display significantly shorter 3′ UTRs but higher expression. Boxplots in the inset demonstrate that the 3′ UTR length of polysome-enriched transcripts was significantly shorter from round to elongating spermatids (Student’s *t*-test and Wilcoxon rank sum test; see Additional file 1: Table S4 for *p* values), but it appears to increase from pachytene spermatocytes to round spermatids, although this is statistically insignificant. **e** Expression profiles of four genes (*Cebpg*, *Eif4h*, *Ubp1*, and *Hip1*), each with multiple transcript isoforms, in late meiotic (pachytene spermatocytes) and haploid (round and elongating) male germ cells. One longer 3′ UTR transcript isoform (in *blue*) and one shorter 3′ UTR isoform (in *red*) with the largest fold changes (*FC*) are shown for each of the four genes. Note that the longer 3′ UTR isoforms, in general, were downregulated, whereas the shorter 3′ UTR isoforms were upregulated. *P* values are based on Student’s *t*-test. *Asterisks* indicate statistically significant *p* values

Significant differences in average 5′ UTR length between RNP- and polysome-enriched mRNAs were only observed in round spermatids (Additional file 1: Table S3; Student’s *t*-test, *p* = 2.0e-6, and Wilcoxon rank sum test, *p* < 2.2e-16), but not in pachytene spermatocytes and elongating spermatids (Additional file 1: Table S3). In contrast, the 3′ UTRs of RNP-enriched transcripts were universally shorter than those of the polysome-enriched ones in all three types of spermatogenic cells analyzed (Additional file 1: Table S4; Student’s *t*-test and Wilcoxon rank sum test, *p* values ranged from 2.2e-16 to 2.3e-3). Moreover, the average 3′ UTR length of RNP-enriched mRNAs became increasingly shorter from pachytene spermatocytes to round and then to elongating spermatids (Fig. 3a; Additional file 1: Table S4). While the average 3′ UTR length of polysome-enriched mRNAs showed a drastic decrease from round to elongating spermatids, it appeared to increase from pachytene spermatocytes to round spermatids (Fig. 3b; Additional file 1: Table S4). In addition, an inverse correlation was observed between the 3′ UTR length and the expression levels, i.e., mRNAs with shorter 3′ UTRs displayed higher expression levels in general (Fig. 3c, d). As an example, we showed the dynamic changes in expression levels of shorter and longer 3′ UTR isoforms of four such genes (*Cebpg*, *Eif4h*, *Ubp1*, and *Hip1*) in RNP and polysome fractions from pachytene spermatocytes to round and elongating spermatids (Fig. 3e). In general, expression levels of longer 3′ UTR isoforms decreased, whereas those with shorter 3′ UTRs increased in both RNP and polysome fractions from pachytene spermatocytes to elongating spermatids. To validate these dynamic changes, we performed quantitative PCR (qPCR) analyses and the results (Additional file 1: Figure S5) were consistent with those from bioinformatic analyses (Fig. 3e).

Together, these data revealed that the overall 3′ UTR length became increasingly shorter from pachytene spermatocytes to round spermatids in both RNPs and polysomes. For genes with multiple transcript isoforms, longer 3′ UTR isoforms were downregulated, whereas shorter 3′ UTR isoforms were upregulated, with round spermatids developing into elongating spermatids.

### RNP-enriched miRNAs preferentially target RNP-enriched mRNAs

**Fig. 4:**
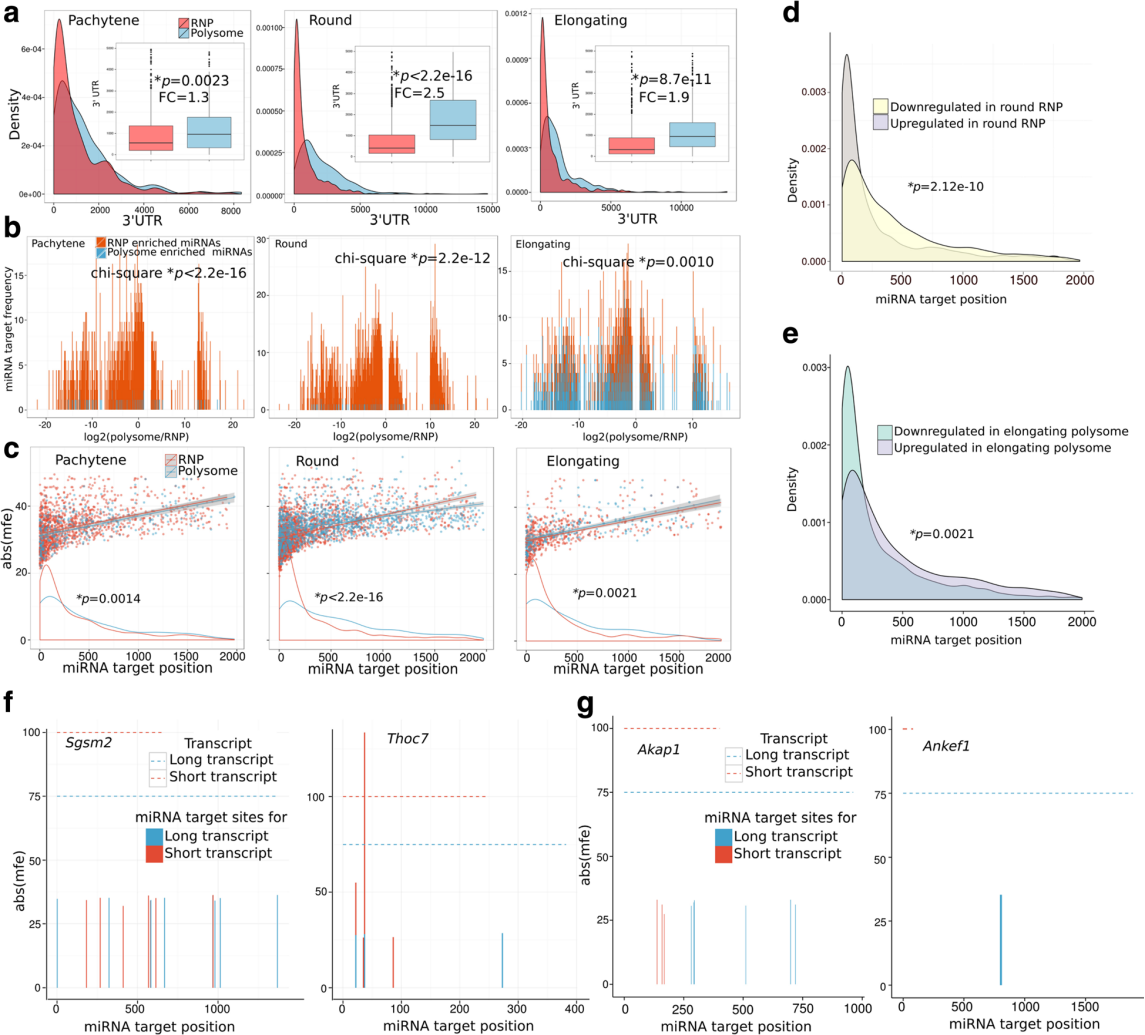
RNP-enriched miRNAs tend to target RNP-enriched mRNAs and the position of miRNA targeting sites correlates with the subcytoplasmic compartmentalization of miRNA targets. **a** Density plots showing the distribution of 3′ UTR length between RNP and polysome fractions in pachytene spermatocytes, round spermatids, and elongating spermatids. The RNP- and polysome-enriched transcripts are defined as those significantly upregulated in RNP or polysome fractions, respectively (Student *t*-test, *p* < 0.1). Boxplots in the inset show the average 3′ UTR lengths in RNP and polysome fractions. Overall, the 3′ UTRs are significantly shorter in RNP fractions than in polysome fractions among all three spermatogenic cell types. The Student’s *t*-test was performed with cross-validation through Wilcoxon rank sum test (*p* = 2.8e-05, 2.2e-16 and 2.2e-16). **b** Histograms showing the relationship between RNP-enriched miRNAs and RNP-enriched mRNAs. The *y-axis* represents the frequency at which miRNAs target the 3′ UTRs of their target mRNAs, while the *x-axis* shows the log_2_ values of ratios of polysome expression levels to RNP expression levels of the target mRNAs (polysome/RNP). RNP-enriched mRNAs are located to the *left* (of zero), whereas those polysome-enriched ones are on the *right* (of zero) along the *x-axis*. **c** Scatter and density plots showing the relationship between miRNA binding sites on the 3′ UTRs of miRNA targets (i.e., mRNAs) and miRNA binding energy. The *y-axis* represents the binding energy of the RNP-enriched miRNAs, whereas the *x-axis* displays the miRNA target position, i.e., the distance between miRNA binding sites and the stop codon. The scatter plots show the distributions of targeting energy versus targeting position of all RNP-enriched (*orange dots*) or polysome-enriched (*blue dots*) miRNAs. The density plots summarize the distribution of the targeting energy versus targeting positions between RNP-enriched (*orange lines*) and polysome-enriched miRNAs (*blue lines*). Note that RNP-enriched miRNAs tend to bind their targets at positions proximal to the stop codon in RNP-enriched mRNAs, whereas those polysome-enriched miRNAs appear to often target the positions distal to the stop codon. The Student’s *t*-test was performed with cross-validation through Wilcoxon rank sum test (*p* = 0.0029, 2.2e-16, and 0.0024). **d** Density plots showing the miRNA target site distribution between RNP up- and downregulated mRNAs from pachytene spermatocytes to round spermatids. The up- or downregulated mRNAs in RNP fractions were compared between pachytene spermatocytes and round spermatids (Student’s *t*-test, *p* < 0.1), and these mRNAs were all those that could be targeted by the miRNAs that were significantly upregulated in RNP fractions from pachytene spermatocytes to round spermatids. Note that the downregulated transcripts tend to contain miRNA target sites more distal to the stop codon (an average of 436 bp), whereas the upregulated RNP transcripts usually have miRNA targeting sites more proximal to the stop codon (an average of 291 bp) (Student’s *t*-test, *p* < 2.2e^−16^). **e** Density plots showing the miRNA target site distribution between polysome up- and downregulated mRNAs from round spermatids to elongating spermatids. The up- or downregulated mRNAs in the polysome fractions were compared between round spermatids and elongating spermatids (Student’s *t*-test, *p* < 0.1), and these mRNAs were all those that could be targeted by the miRNAs that were significantly upregulated in the polysome fractions from round spermatids to elongating spermatids. Note that the downregulated transcripts tend to contain miRNA target sites more distal to the stop codon, whereas the upregulated polysome transcripts usually have miRNA targeting sites more proximal to the stop codon. **f**, **g** The shorter (upregulated in *red*) and longer (downregulated in *blue*) 3′ UTR transcript isoforms of *Sgsm2*, *Thoc7*, *Akap1*, and *Ankef1* are presented as *dotted lines*. The absolute binding energy of upregulated miRNAs in round spermatid RNP fractions is shown on the *y-axis*; the *x-axis* shows the miRNA target positions, i.e., the distance between the miRNA binding sites to the stop codon. The upregulated RNP miRNAs appear to target more distal sites in the longer 3′ UTR transcripts compared to the shorter 3′ UTR isoforms in the round spermatid RNP fractions. *Asterisks* indicate statistically significant *p* values

The fact that the 3′ UTR length becomes increasingly shorter in RNPs than in polyribosomes from pachytene spermatocytes to round spermatids suggests a mechanism through which transcripts with shorter 3′ UTRs are selectively recruited into RNPs, whereas those with longer 3′ UTRs stay in or relocate to the polysome fractions. To test this hypothesis, we first investigated whether RNP-enriched mRNAs are preferentially targeted by RNP-enriched miRNAs and whether polysome-enriched miRNAs tend to bind polysome-enriched mRNAs. By comparing the mRNA 3′ UTR length distribution between RNP and polysome fractions in each cell type, we found that 3′ UTRs of RNP-enriched transcripts were significantly shorter than those of polysome-enriched transcripts (Student’s *t*-test, *p* values ranged from 2.2e-16 to 2.3e-3; Fig. 4a).

The preferential enrichment of miRNAs in RNPs prompted us to explore the possibility that miRNAs may function to recruit their target mRNAs into RNPs. To test this, we analyzed the ability of the RNP-enriched miRNAs to target the 3′ UTRs of RNP-enriched mRNAs through their seed sequences (2–7 nucleotides) using RNAhybrid [42]. We found that 74% and 54% of the RNP-enriched mRNAs were targeted by RNP-enriched miRNAs in both pachytene spermatocytes and round spermatids, respectively (Additional file 1: Tables S5 and S6). A strong positive correlation was observed between mRNAs and their targeting miRNAs enriched in RNPs of both pachytene spermatocytes (χ^2^ test, *p* = 2.2e-16) and round spermatids (χ^2^ test, *p* = 2.3e-12). The targeting frequency of RNP-enriched miRNAs was significantly greater in RNP-enriched mRNAs than in polysome-enriched mRNAs in both pachytene spermatocytes and round spermatids (Fig. 4b). Interestingly, in elongating spermatids, the targeting frequency of both RNP-enriched and polysome-enriched miRNAs appeared to increase, and mRNAs enriched in both fractions were targeted (Fig. 4b; Additional file 1: Tables S7 and S8). This drastic change coincides with the shifting of a substantial proportion of miRNAs from RNPs to polysomes in elongating spermatids, as shown in Fig. 2c.

Since the number of polysome-enriched miRNAs is much smaller than that of RNP-enriched miRNAs, the relationship between polysome-enriched miRNAs and mRNAs was less clear (Fig. 4b). Therefore, we further studied the binding energy of polysome-enriched miRNAs and unexpressed miRNAs (100 miRNAs undetectable in any of the three spermatogenic cell types as control) in the 3′ UTRs of polysome-enriched mRNAs using RNAhybrid. The binding capacity of polysome-enriched miRNAs was significantly higher than that of unexpressed miRNAs in all three spermatogenic cell types (Additional file 1: Figure S6a). Similarly, RNP-enriched miRNAs displayed much higher binding energy than the unexpressed miRNAs (data not shown). These data suggest that the partnership between RNP-enriched miRNAs and RNP-enriched mRNAs, or between polysome-enriched miRNAs and polysome-enriched mRNAs, is not random, but a specific event.

Together, these data support our hypothesis that miRNAs may help recruit and stabilize mRNAs to the RNPs from pachytene spermatocytes to round spermatids. Once round spermatids develop into elongating spermatids, a significant proportion of miRNAs exit from RNPs and become enriched in polysomes.

### RNP- and polysome-enriched miRNAs tend to bind regions proximal and distal to the stop codon in 3′ UTRs of RNP- and polysome-enriched mRNAs, respectively

Although RNP-enriched miRNAs appeared to act mostly on RNP-enriched mRNAs in general, they could, in theory, target polysome-enriched mRNAs as well. For example, 172 and 485 mRNAs, although targetable by RNP-enriched miRNAs, were nevertheless enriched in the polysome fractions in pachytene spermatocytes and round spermatids, respectively (Additional file 1: Tables S5 and S6). To identify the differences between these two populations of mRNAs, we performed random forest data mining [43, 44] and uncovered that these polysome-enriched mRNAs typically contained miRNA binding sites that were ~461 nucleotides downstream of the stop codon, whereas the miRNA binding sites for the RNP-enriched mRNAs were usually closer to the stop codon (~365 nucleotides) in pachytene spermatocytes (Additional file 1: Table S5). Similar phenomena were observed in round and elongating spermatids, where RNP-enriched miRNAs targeted mRNAs containing a proximal miRNA targeting sites (311 nucleotides and 346 nucleotides), whereas those target mRNAs enriched in polysomes tended to contain distal miRNA binding sites (552 nucleotides and 452 nucleotides) (Additional file 1: Table S6 and S7; Student’s *t*-test, *p* values 2.2e-16 and 2.1e-3, respectively; Wilcoxon rank sum test, *p* values 2.1e-3 and 2.4e-3, respectively). By plotting the miRNA binding energy (absolute value of minimum free energy) against the miRNA targeting position in 3′ UTRs, we noticed higher binding energy when RNP-enriched miRNAs target the RNP-enriched mRNAs via the proximal sites compared to binding the polysome-enriched mRNAs using the distal binding sites (Fig. 4c).

The relationships between polysome-/RNP-enriched miRNAs and mRNAs were further analyzed using both RNAhybrid [42] and TargetScan [45]. The RNAhybrid-based miRNA–mRNA binding assays showed that RNP-enriched miRNAs tend to target regions proximal to the stop codon, whereas polysome-enriched miRNAs prefer binding sites distal to the stop codon in 3′ UTRs of mRNAs expressed in three spermatogenic cell types, including pachytene spermatocytes and round and elongating spermatids (Additional file 1: Figure S7a). Consistently, the TargetScan-based miRNA target identification revealed that the targeting sites of miRNAs in RNP-enriched mRNAs are closer to the stop codon than those in polysome-enriched mRNAs in both pachytene spermatocytes and round spermatids (Additional file 1: Figure S7b). An opposite relationship was observed in elongating spermatids, probably reflecting the massive release of the formerly RNP-enriched mRNAs due to the demise of the chromatid body and more efficient translation in the cytoplasm during the elongation stage of spermatogenesis (Additional file 1: Figure S7b).

To evaluate the specificity of these observations, we further studied the binding sites of polysome-enriched miRNAs and unexpressed miRNAs (100 miRNAs undetectable in any of the three spermatogenic cell types) in the 3′ UTRs of polysome-enriched mRNAs using RNAhybrid. The polysome-enriched miRNAs appeared to prefer distal sites in the 3′ UTRs of polysome-enriched mRNAs compared to the unexpressed miRNAs (Additional file 1: Figure S6b). Similarly, the RNP-enriched miRNAs tended to bind regions closer to the stop codon than those bound by unexpressed miRNAs in the 3′ UTRs of RNP-enriched mRNAs (data not shown). Thus, the preferential binding of RNP- and polysome-enriched miRNAs to proximal and distal sites in the 3′ UTRs of RNP- and polysome-enriched mRNAs, respectively, is not random, but a specific event.

These data suggest that miRNAs may recognize mRNAs containing the proximal binding sites and recruit them to the RNPs for stabilization and translational suppression; meanwhile, mRNAs containing the distal binding sites may shift from the RNPs to polysomes for translation and/or degradation. Consistent with earlier reports [46–48], these data support a positional effect of miRNA targeting sites on mRNA fate.

### Newly synthesized miRNAs appear to guide the redistribution of mRNA subcytoplasmic compartmentalization in pachytene spermatocytes and round spermatids

If miRNAs truly function to control mRNA compartmentalization and thus their fates, the changes in mRNAs and their compartmentalization should correlate with the changes in miRNAs in the three types of spermatogenic cells. To test this, we analyzed the dynamic changes in mRNA and miRNA contents in the RNP fractions from pachytene spermatocytes to round spermatids. In the RNP fractions from pachytene spermatocytes to round spermatids, 1068 transcripts were significantly downregulated, whereas 909 transcripts were drastically upregulated at the same time (Additional file 1: Table S9). Correspondingly, 216 miRNAs were upregulated, whereas only one miRNA was downregulated (Additional file 1: Table S9). Interestingly, statistical analyses indicated a significant positive correlation between the upregulated miRNAs and the upregulated or downregulated mRNAs in RNPs (Additional file 1: Table S9; *X* ^*2*^ test, *p* = 1.6e-12). From pachytene spermatocytes to round spermatids, the downregulated mRNAs in RNPs were mostly those with longer 3′ UTRs and distal miRNA targeting sites; in contrast, the upregulated mRNAs in RNPs were mostly those with shorter 3′ UTRs containing proximal miRNA binding sites (Fig. 4d). A similar phenomenon was noted from round to elongating spermatids, with upregulated mRNAs in polysomes containing shorter 3′ UTRs and proximal miRNA binding sites and downregulated mRNAs in polysomes having longer 3′ UTRs and distal miRNA binding sites for the miRNAs shifted from RNPs to polysomes (Fig. 4e; Additional file 1: Table S10). To further validate this finding, we analyzed two genes, *Sgsm2* and *Thoc7*, each with two isoforms: one with longer and the other with short 3′ UTRs (Fig. 4f). Upregulated miRNAs tended to target the distal sites of the long 3′ UTR transcripts, coinciding with their decreased expression in RNPs. Meanwhile, upregulated miRNAs targeted the proximal sites in the shorter 3′ UTR transcripts, correlating with upregulation of the shorter 3′ UTR transcripts in the RNP fraction of round spermatids (Fig. 4f). Similarly, both *Akap1* and *Ankef1* expressed two isoforms, and upregulated miRNAs targeted the distal sites of the longer 3′ UTR isoforms that were downregulated and the proximal sites in the short 3′ UTR isoforms that showed upregulated expression in the polysome fractions of elongating spermatids (Fig. 4g). These correlations suggest that newly synthesized miRNAs may help localize their target mRNAs to the RNPs if the newly transcribed mRNAs contain shorter 3′ UTRs and the proximal binding sites. At the same time, those RNP-localized mRNAs containing distal miRNA binding sites with relatively longer 3′ UTRs will be targeted by the newly synthesized miRNAs and removed from RNPs, leading to a relocation from RNPs to polysomes for translation or degradation. The miRNAs shifted from RNPs to polysomes may target the distal sites of longer 3′ UTR transcripts in polysomes, leading to translation or degradation.

### *Drosha* inactivation leads to global destabilization and aberrant cytoplasmic compartmentalization

**Fig. 5:**
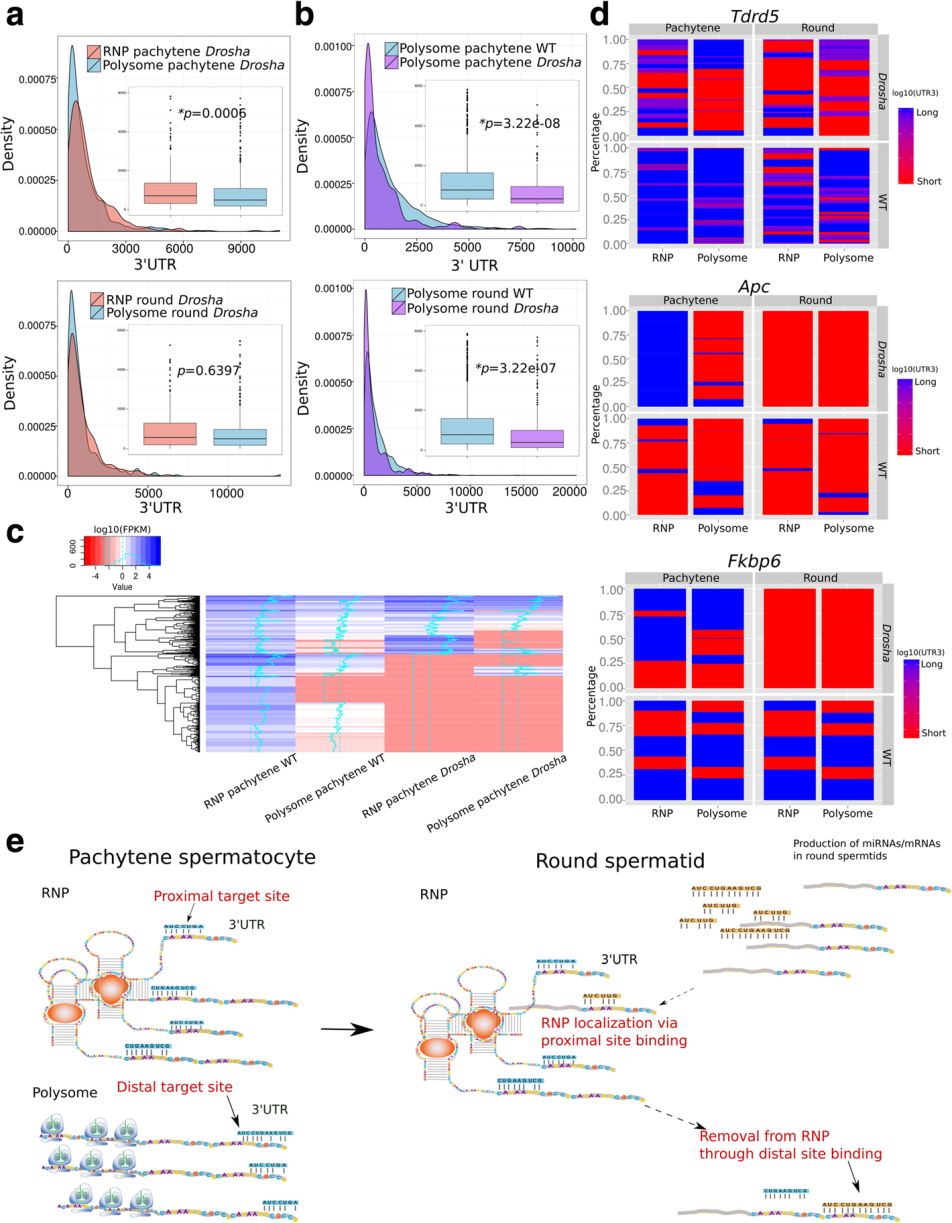
Changes in mRNA stability and compartmentalization in *Drosha*-null spermatogenic cells. **a** Density plots showing the distribution of 3′ UTR length of RNP- or polysome-enriched mRNAs in *Drosha*-null pachytene spermatocytes (“*RNP pachytene Drosha*” or “*Polysome pachytene Drosha*” in *upper panel*)) and round spermatids (“*RNP round Drosha*” or “*Polysome round Drosha*” in *lower panel*). Boxplots in the insets show the average size of 3′ UTRs of RNP- and polysome-enriched mRNAs in *Drosha*-null pachytene spermatocytes (*upper panel*) and round spermatids (*lower panel*). Student’s *t*-test was performed. **b** Density plots showing the distribution of 3′ UTR length of polysome-enriched mRNAs in wild-type (*WT*) or *Drosha*-null pachytene spermatocytes (*upper panel*) and round spermatids (*lower panel*). Boxplots in the insets demonstrate the average 3′ UTR size in polysome-enriched mRNAs in wild-type and *Drosha*-null pachytene spermatocytes (*upper panel*) and round spermatids (*lower panel*). Student’s *t*-test was performed. **c** Heatmap showing levels of RNP- and polysome-enriched mRNAs in wild-type or *Drosha*-null pachytene spermatocytes and round spermatids. **d** Changes in expression patterns and cytoplasmic compartmentalization of multiple isoforms of three genes (*Tdrd5*, *Apc*, and *Fkbp6*) in the RNP and polysome fractions in pachytene spermatocytes and round spermatids. **e** The proposed mechanism through which miRNAs control mRNA compartmentalization by recruiting shorter 3′ UTR transcripts into the RNPs and removing longer 3′ UTR transcripts from the RNPs. In pachytene spermatocytes, RNP-enriched mRNAs generally have shorter 3′ UTRs than polysome-enriched mRNAs, and the RNP-enriched mRNAs are associated with RNP-enriched miRNAs at sites proximal to the stop codon in 3′ UTRs, whereas polysome-enriched mRNAs tend to be bound by miRNAs at sites distal from the stop codon in the 3′ UTRs. When pachytene spermatocytes develop into round spermatids, new miRNAs are produced which recruit shorter 3′ UTR mRNAs into RNPs by binding the proximal sites; meanwhile, mRNAs containing distal binding sites, which are usually those with longer 3′ UTRs, are removed from RNPs. In this manner, the RNP fraction becomes gradually enriched with shorter 3′ UTR transcripts. When round spermatids develop into elongating spermatids, a significant proportion of RNP-enriched miRNAs shift out of RNPs and their target mRNAs also shift to polysomes for translation. *Asterisks* indicate statistically significant *p* values

*Drosha* is essential for miRNA production and *Drosha* inactivation in spermatogenic cells leads to germ cell depletion and male infertility due to oligo-astheno-teratozoospermia [49–52]. As described above, our bioinformatic analyses suggest an active role of miRNAs in compartmentalizing their target mRNAs based on the 3′ UTR length. To further test this notion, we analyzed *Drosha*-null spermatogenic cells and examined the effects of miRNA deficiency on the compartmentalization and expression levels of mRNAs. We previously have demonstrated that up to 80% of miRNAs were depleted in *Drosha*-null pachytene spermatocytes and round spermatids [49]. Now our analyses of 3′ UTRs using SpliceR revealed that the average 3′ UTR length was much shorter in polysomes than in RNPs (Fig. 5a), a pattern opposite that in wild-type cells, where the 3′ UTR length of RNP transcripts is much shorter than that of polysome transcripts (Fig. 3). Moreover, the average 3′ UTR length of mRNAs enriched in polysomes was much shorter in *Drosha*-deficient pachytene spermatocytes and round spermatids than in wild-type counterparts (Fig. 5b), suggesting that those shorter 3′ UTR transcripts fail to localize to RNPs when miRNAs are largely depleted or aberrantly expressed. The levels of the RNP-enriched mRNAs in wild-type spermatogenic cells were drastically downregulated in *Drosha* conditional knock-out (cKO) cells, supporting the notion that these mRNAs fail to localize to RNPs and get stabilized, leading to degradation and severe downregulation in the *Drosha* cKO cells (Fig. 5c). Transcripts in RNPs and polysomes were massively downregulated in the *Drosha* cKO cells compared to wild-type cells, suggesting that mRNAs are unstable in both RNPs and polysomes when miRNAs are deficient (Fig. 5c). We then looked into three genes (*Tdrd5*, *Apc*, and *Fkbp6*) that are expressed as multiple longer or shorter 3′ UTR isoforms (Fig. 5d). In general, the shorter 3′ UTR isoforms were drastically upregulated in the polysomes in *Drosha* cKO cells, especially in pachytene spermatocytes, and the normal distribution patterns were drastically altered. However, the upregulated short 3′ UTR transcripts are likely different from the ones in wild-type cells because the overall levels of transcripts were downregulated (Fig. 5c). The aberrant distribution of longer or shorter 3′ UTR transcript isoforms and their cytoplasmic compartmentalization support our notion that miRNAs appear to control mRNA fate by compartmentalizing mRNAs based on the 3′ UTR length.

## Discussion

Cytoplasmic compartmentalization is known to be an important mechanism through which RNA stability and function are regulated [6, 53, 54]. Both large and small RNAs as well as RNA-binding proteins are abundant in RNPs, which has led to the hypothesis that RNPs play a critical role in regulating mRNA fate [55]. By profiling mRNAs relatively enriched in RNPs or polysomes, many studies have shown that mRNAs accumulated in RNPs tend be more stable and translationally suppressed. In contrast, mRNAs associated with polysomes represent those being actively translated in the cell [28].

In the testis, selective enrichment of transcripts in RNPs is a continuous process which occurs in late meiotic (in pachytene spermatocytes) and lasts until early haploid (in round spermatids) phases [28, 33]. An obvious question remains: what is the underlying mechanism? Our thorough transcriptomic analyses uncovered a potential miRNA-mediated mechanism for the RNP enrichment of mRNAs, especially those with shorter 3′ UTRs. Specifically, when pachytene spermatocytes develop into round spermatids, the newly synthesized mRNAs, if they can be targeted by newly synthesized miRNAs at the proximal sites, would localize to RNPs. In contrast, those with distal miRNA binding sites would not localize to RNP and thus become subject to either translation or degradation. Existing RNP mRNAs, if targeted by the newly synthesized miRNAs at the distal sites, would be removed from RNPs and shift to polysomes for translation or degradation (Fig. 5e). In this manner, waves of newly synthesized miRNAs could target an increasing number of transcripts with shorter 3′ UTRs to RNPs and remove more and more transcripts with longer 3′ UTRs from RNPs, leading to an enrichment of shorter 3′ UTR transcripts in RNPs compared to polysomes (Additional file 2: Movie S1).

Our data strongly support this potential mechanism. First, miRNAs are mostly enriched in RNPs from pachytene spermatocytes to round spermatids, in which numerous mRNAs are kept in non-translational or translationally suppressive states; ~37% of RNP-enriched miRNAs shift from RNPs to polysomes in elongating spermatids where an increasing number of mRNAs undergo translation for sperm assembly. Second, RNP-enriched miRNAs preferentially target RNP-enriched mRNAs and the miRNA binding sites are closer to the stop codon compared to polysome-enriched mRNAs that can also be targeted by the same sets of miRNAs, but through targeting sites more distal to the stop codon. Third, newly synthesized miRNAs positively correlate with upregulation of a subset of newly synthesized mRNAs, which are mostly transcripts with shorter 3′ UTRs containing the proximal miRNA binding sites; conversely, the new miRNAs negatively correlate with transcripts that are shifting out of RNPs to polysomes and contain the distal miRNA binding sites. The proposed sequence of events, as illustrated in Fig. 5e and Additional file 2: Movie S1, represents the most likely mechanism through which miRNAs control mRNA cytoplasmic compartmentalization and thus their fate during spermiogenesis.

Since developing male germ cells cannot be cultured for an extended period of time, it is not feasible to recapitulate all the events in an in vitro system. However, *Drosha* cKO cells provided us with an opportunity to test whether this hypothetical mechanism works in vivo. Indeed, ablation of miRNAs disrupts subcytoplasmic compartmentalization, e.g., shorter 3′ UTR transcripts fail to be sequestered in RNPs, leading to transcript degradation and/or possibly precocious translation. Earlier reports have linked pachytene piRNAs to the control of mRNA stability during the haploid phase of spermatogenesis [56, 57]. Therefore, it would be interesting in the future to examine the relationship between the transcripts targeted by piRNA-guided bulk degradation and transcripts that are under the control of miRNAs in the context of their subcytoplasmic compartmentalization and 3′ UTR length. Given that numerous RNA-binding proteins (RBPs) have been shown to regulated mRNA fate by binding the 3′ UTRs of their target transcripts, it remains interesting to study the relationship between miRNAs and RBPs. Our preliminary analyses of the expression profiles of 73 RBPs known to be abundantly expressed in male germ cells in late meiotic and haploid phases showed that the RBP-binding sites are often close to miRNA-binding sites (Additional file 1: Figure S8), supporting the notion that miRNA and RBPs may interact and jointly control mRNA fate. More future studies are needed to gain more insights. Nevertheless, all our data strongly support a critical role of miRNAs in the control of mRNA compartmentalization and mRNA fate (Fig. 5e).

RNPs are known to be enriched in both large and small RNAs, as well as RNA binding proteins, and often associate with sub-cytoplasmic domains, including stress granules, processing bodies (p-bodies), and exosomes, in somatic cells [55, 58–61]. In male germ cells, RNPs appear to be associated with the structure called intermitochondrial cement, or nuage, in both mitotic and meiotic spermatogenic cells (i.e., spermatogonia and spermatocytes) [62, 63]. After meiosis, male germ cells enter the haploid phase; in round spermatids, RNPs are confined to a structure called the chromatoid body (CB), which is a granular structure constantly moving along the nuclear membrane and frequently contacting the nuclear pores [64–66]. It has been postulated that the CBs can collect transcripts exported from the nuclei through the nuclear pores for post-transcriptional processing, storage, transport, and stabilization. Interestingly, the CBs disappear upon spermatid elongation and the RNPs are believed to be less prominent in elongating and elongated spermatids because the processing of haploid transcripts has completed and translation becomes the main theme because transcription ceases when nuclear condensation and elongation commence in step 9 spermatids [27, 28].

The dynamic changes in the morphology of the RNP-associated structures in later meiotic and haploid male germ cells appear to correlate well with the changes in RNP-enriched RNA contents based on our data. In late pachytene spermatocytes where the RNPs are associated with nuage or intermitochondrial cement, the number of mRNAs enriched in RNPs is much greater than that enriched in polysomes, suggesting enhanced translational suppression of mRNAs at the late pachytene stage of meiosis. Moreover, preferential enrichment of miRNAs in RNPs rather than in polysomes suggests a potential role of miRNAs in regulating mRNA enrichment in RNPs. In round spermatids where RNPs are confined to the CBs, while the number of RNP-enriched mRNAs remains greater than that of polysome-enriched ones, a significant change in 3′ UTR length occurs, i.e., the 3′ UTR length becomes increasingly shorter in RNP-enriched transcripts from pachytene spermatocytes to round spermatids. Interestingly, the average 3′ UTR length, meanwhile, does not decrease significantly because many longer 3′ UTR transcripts appear to be released from RNPs and accumulated in polysomes for translation. Intriguingly, newly produced miRNAs are almost all enriched in RNPs, as are the newly synthesized shorter 3′ UTR mRNAs containing proximal binding sites for those new miRNAs. These data strongly suggest a cause–effect relationship between miRNAs and the compartmentalization of mRNAs in RNPs. When round spermatids develop into elongating spermatids, the task of enriching short 3′ UTR transcripts is completed once transcription ceases in step 9 spermatids; at the same time, the special RNP compartment, i.e., the CB, gradually disintegrates, and consequently miRNAs and their targets are released into the cytoplasm and loaded onto polysomes for quick translation followed by degradation. The enhanced translational efficiency highly likely results from shorter 3′ UTRs, which allow for binding by much fewer miRNAs and RBPs, thus increasing efficiency. Taken together, our data, for the first time, connect morphological changes of RNP structures to changes in RNA contents in late meiotic and haploid phases of spermatogenesis.

Post-transcriptional regulation, in large part, is achieved through binding of 3′ UTRs of mRNAs by RBPs and sncRNAs (e.g., miRNAs, endo-siRNAs, pachytene piRNAs, or piRNA-like small RNAs) [7, 28, 58, 61]. Therefore, regulation of 3′ UTR length can be an efficient way to control mRNA fate. Indeed, numerous studies have shown that longer 3′ UTRs can be regulated by more RBPs and sncRNAs, thus allowing higher orders of regulation of gene expression [5, 20, 24, 26]. Interestingly, brain transcriptome appears to be enriched with longer 3′ UTR transcripts [22, 58, 61]. In contrast, previous transcriptomic studies have demonstrated that the testis, unlike brain, contains a transcriptome with abundant shorter 3′ UTR transcripts [26]. The transcriptome of haploid male germ cells, i.e., spermatids, is highly enriched with transcripts of shorter 3′ UTRs compared to the transcriptomes of mitotic and meiotic spermatogenic cells (i.e., spermatogonia and spermatocytes) [20, 26]. Given the fact that the longer or shorter 3′ UTR transcripts are mostly derived from genes with multiple transcript isoforms, it has been postulated that alternative polyadenylation (APA) represents the most likely underlying mechanism [5]. However, the identity of such APA factors and their actions remain largely unknown. Interestingly, recent reports discovered that elimination of longer 3′ UTR transcripts can lead to relative enrichment of the shorter 3′ UTR transcripts, and the selective degradation of longer 3′ UTR transcripts is achieved by UPF1/UPF2-mediated nonsense mRNA decay (NMD) pathway [15–17]. This finding suggests that both APA and NMD pathways can act either jointly or independently to achieve 3′ UTR shortening or lengthening. Here, our data add another player into the machinery of 3′ UTR length control, i.e., miRNAs. Depending on the binding sites in 3′ UTRs, miRNAs appear to be able to recruit mRNAs with proximal binding sites, which are usually transcripts with shorter 3′ UTRs, to the RNPs. In contrast, since longer 3′ UTR transcripts often contain distal binding sites, these transcripts, once bound by miRNAs, will either be released from the RNPs or assume non-RNP localization in the cytoplasm, rendering themselves to translation and/or degradation. Through waves of miRNA production from late pachytene spermatocytes to round spermatids, mRNAs that need to be translationally suppressed, which mostly participate in late spermiogenesis, are gradually recruited and become highly enriched in RNPs; upon spermatid elongation, the CBs degenerate and those RNP-enriched mRNAs are released for efficient translation to ensure proper sperm assembly during the final several steps of sperm assembly. In the context of 3′ UTR shortening during spermiogenesis, miRNAs apparently participate in this process by recruiting shorter 3′ UTR transcripts to the RNPs for stabilization and promoting the release of longer 3′ UTR transcripts from RNPs or preventing them from entering RNPs; more importantly, miRNAs recognize 3′ UTR length based on the proximity of their binding sites to the stop codon. It remains interesting to study in the future how miRNAs interact with NMD or APA factors and coordinate the selective depletion of longer 3′ UTR transcripts.

Taken together, we have conducted the first ever comprehensive RNA-Seq-based transcriptomic profiling and defined dynamic changes in not only expression levels but also cytoplasmic compartmentalization of both mRNAs and sncRNAs during a specific window of spermatogenesis (i.e., late meiotic to early and mid-haploid phases), which are known to be subjected to profound post-transcriptional regulation (numerous mRNAs are synthesized without translation until a much later time). Our bioinformatic analyses led to the discovery of an elegant mechanism through which miRNAs control cytoplasmic compartmentalization of mRNAs based on the 3′ UTR length in male germ cells. This finding was only possible using next-gen genomics and bioinformatics analyses, and our proposed mechanism may well be applicable to other cells that use 3′ UTR length control as a means of post-transcriptional regulation. It is also noteworthy that given the potential complex interactions among multiple 3′ UTR-binding factors (e.g., RBPs, miRNAs, piRNAs, endo-siRNAs, etc.), our proposed model (Fig. 5e) may represent a simplified one, which needs to be further validated by additional experiments in the future.

## Conclusions

miRNAs appear to control cytoplasmic compartmentalization of mRNAs based on the 3′ UTR length in male germ cells. Specifically, transcripts with longer 3′ UTRs tend to contain distal miRNA binding sites and are thus targeted to polysomes for translation followed by degradation. In contrast, those with shorter 3′ UTRs only possess proximal miRNA binding sites and thus tend to be targeted into the RNPs for enrichment and delayed translation.

## Methods

### Animals

All mice used in this study were on the C57BL/6 J background, and housed under specific pathogen-free conditions in a temperature- and humidity-controlled animal facility at the University of Nevada, Reno. The male germ cell-specific *Drosha* cKO mice were described previously [49].

### Purification of spermatogenic cells

Pachytene spermatocytes and round and elongating/elongated spermatids were purified from adult mouse testes using the STA-PUT method [34]. BSA gradients (0.5–4%) were prepared in EKRB buffer (catalog number K-4002, Sigma), supplemented with sodium bicarbonate (1.26 g/L), L-glutamine (0.29228 g/L), penicillin and streptomycin mix (Thermo-Fisher, 10,000 U/L), MEM non-essential amino acids (Thermo-Fisher, 1 ml 100×/L), MEM amino acids (20 ml 50×/L), and cycloheximide (100 ng/ml), pH7.2–7.3. Eight testes were pooled each time for cell purification. After being removed and decapsulated, testes were placed into 10 ml of EKRB buffer containing 5 mg collagenase (Sigma) for a 12-min digestion at 32 °C to disperse the testicular cells. Once dispersed, the testicular cells were washed three times using EKRB buffer followed by trypsin digestion by incubation in 10 ml EKR buffer containing trypsin (Sigma; 0.25 mg/ml) and DNase I (Sigma; 20 μg/ml) at 37 °C for 12 min with occasional pipetting to facilitate cell dispersion. Fully dispersed testicular cells were washed three times followed by centrifugation and re-suspension in 10 ml of 0.5% BSA. The cell suspension was passed through 50 μm filters and the filtrate was saved for loading onto the STA-PUT apparatus for sedimentation. After 3 h sedimentation at 4 °C, fractions were collected from the bottom of the sedimentation chamber. A total of 30 fractions of 15 ml each were collected. After centrifugation, the supernatants were removed and the cells in each fraction were re-suspended and the cell purity was determined by microscopy examination based on cell morphology, as described previously [34]. Fractions containing the same cell types were pooled followed by centrifugation to collect purified pachytene spermatocytes, round spermatids, and elongating/elongated spermatids. Purity was estimated based on cell morphology as described [67].

### RNP and polysome fractionation

We fractionated the purified spermatogenic cells into RNP, monoribosome, and polyribosome fractions using a continuous sucrose gradient ultracentrifugation method, as described [33]. In brief, a continuous sucrose gradient (15–50%) was prepared by carefully overlaying 15% sucrose onto 50% sucrose followed by diffusing for 3 h at 4 °C. The 15 and 50% sucrose solutions were prepared in a lysis buffer (containing 150 mM potassium acetate, 5 mM magnesium acetate, 2 mM DTT, protease inhibitor cocktail (Sigma, 1×), RNase inhibitor cocktail (Sigma, 1×), cycloheximide (100 ng/ml), and 50 mM HEPES, pH 7.5). Freshly purified pachytene spermatocytes, round spermatids, and elongating/elongated spermatids were homogenized in the lysis buffer freshly supplemented with 0.5% Triton X-100 and 0.25 M sucrose. The homogenates were centrifuged at ~500 g for 15 min at 4 °C to remove tissue debris, unbroken cells, and nuclei. The supernatant was loaded onto the continuous 15–50% sucrose gradient followed by centrifugation at 150,000 g (35,000 rpm) for 3 h at 4 °C. A tiny hole was gently punched in the bottom of the tubes for fraction collection. Twenty-four 500-μl fractions were collected followed by UV spectrometer measurement for OD254, and RNAs in RNP (fractions 1–4), monosome (fractions 5–15), and polysome (fractions 16–22) fractions were used for RNA-Seq and qPCR analyses (Fig. 1).

### RNA-Seq

Large RNAs were isolated using the AquaRNA RNA Purification Kit (catalog number 5001MT, Mo Bi Tec, Inc.), whereas small RNAs were prepared using the mirPremier™ microRNA Isolation Kit (catalog number SNC10, Sigma). The mRNA and sncRNA libraries were constructed using the TruSeq Stranded Total RNA Library Prep Kit (catalog number RS-122-2201, Illumina). Sequencing was conducted using Hi-Seq 2000 sequencers with SE50 at the Genomics Microarray Core in UT Southwestern Medical Center (Dallas, TX, USA) and with PE50 at BGI (Davis, CA, USA).

### Bioinformatic analyses of mRNA-Seq data

The FASTX-Toolkit was used to remove adaptor sequences and low quality reads from the sequencing data. To identify all the transcripts, we used TopHat2 and Cufflinks to assemble the sequencing reads based on the UCSC MM9 mouse genome [68]. The differential expression analyses were performed by Cuffdiff [68]. The sequencing depth and mapping rate are shown in Additional file 1: Table S11. The global statistics and quality controls are described in Additional file 1: Figure S9. A total of 96,896 transcripts were assembled, among which a total of 69,013 transcripts were annotated as mRNAs.

We chose 24 endogenous mRNAs from a list of those known to display equal distribution between RNP and polysome fractions during spermatogenesis in previous studies [33]. We performed qPCR and showed that they are indeed equally distributed (Additional file 1: Figure S2). Therefore, we used these 24 endogenous mRNAs as internal controls and normalized the sequencing reads from RNP and polysome fractions of the three spermatogenic cell types using linear regression as described [40]. Geometric median normalization and quantile normalization were also performed to compare with the linear regression normalization approach [40]. FPKM was set at 1 as the threshold to minimize false positives in downstream analyses.

The UTR and alternative splicing analyses were performed using the SpliceR pipeline [15, 41]. In brief, potential coding DNA sequences (CDS) were first determined by SpliceR, and both 5′ and 3′ UTR sequences were then extracted for UTR length analyses. To validate and correct the ends of the 3′ UTR sequences identified by SpliceR, we downloaded the mouse testis polyadenylation site sequencing (PAS) data from the GEO database at the NCBI (accession GSM747485) and compared these with SpliceR-derived 3′ UTR sequences. R script was used to assist the data mining. The log_10_(3′ UTR length) values followed normal distribution (Additional file 1: Figure S10).

### In silico miRNA target prediction

The 3′ UTR sequence for each RNA isoform was extracted using an in-house R script based on the SpliceR analyses. RNAhybrid [42] was used to identify both canonical and non-canonical targets of miRNAs (RNAhybrid –c –s 3utr_human –p 0.1 -f 2,7). Given that the noncanonical and canonical targets identified using RNAhybrid were almost the same, we only present the results of canonical targets based on the matching seed sequence (second to seventh nucleotides). TargetScan [45] was also used to identify miRNA binding sites in the 3′ UTRs of their target mRNAs. We used the high confidence miRNA binding sites in the TargetScan database (http://www.targetscan.org/mmu_71/). The output results were imported into R as a data frame for downstream analyses.

### sncRNA-Seq data analysis

The FASTX-Toolkit was used to remove adaptor sequence and low quality reads from the sequencing data. We then aligned the sncRNA-seq reads with the FASTA file containing all known sncRNA sequences using bowtie 2 (bowtie2 –-end-to-end –N 1 –L 16 –i S,0,0.2) [69]. Bowtie2 is usually used as a genome aligner; however, in our hands it achieved an R^2^ value of 0.97 for small RNA alignments with known small RNA species after we adjusted the parameters. The aligned reads were counted by featureCounts [70]. This new pipeline ran much faster than the traditional methods, which align the reads with the genome, and it took only 30 min for 5 gigabits of data. The count data were imported into R package Deseq2 for normalization and statistical analysis [71].

### qPCR-based validation

Semi-quantitative and real-time quantitative PCR analyses were performed as described [15, 50, 67, 72, 73]. In brief, equal amounts of RNA were reverse-transcribed using SuperScript II (Thermo fisher catalog number 18064014) followed by 15–20 cycles of PCR using the GoTaq Green Master Mix (Promega catalog number M7123). The PCR products were then visualized on 2% agarose gels. qPCR analyses were performed using the SYBR Green PCR Master Mix (Thermo fisher catalog number 4309155) on a qPCR system (Applied Biosystems 7900HT).

### Statistical analyses

Both Student’s *t*-test and Wilcoxon rank sum test (a non-parametric or distribution-free test) were used for statistical analyses. The majority of the data followed a lognormal distribution. Student’s *t*-test was also performed on the logarithm data.

## Additional files

Additional file 1: Contains supplementary figures and tables: **Figures S1**–**S10.** and **Tables S1**–**S11.** (PDF 30123 kb)

Additional file 2: A flash movie showing the step-wise actions of miRNAs in targeting mRNAs to the RNPs based on the 3′ UTR length in pachytene spermatocyte, round spermatids, and elongating spermatids. (MP4 417 kb)
