# Supplementary material for: MicroRNAs control mRNA fate by compartmentalization based on 3′ UTR length in male germ cells
Source: Genome Biol. 2017 Jun 15;18:105. doi: 10.1186/s13059-017-1243-x (PMC5471846; doi:10.1186/s13059-017-1243-x)
Supplement: Supplementary file 1 — Contains supplementary figures and tables: Figures S1–S10. and Tables S1–S11. (PDF 30123 kb) [file 13059_2017_1243_MOESM1_ESM.pdf]

Supplemental Information

miRNAs control mRNA fate by compartmentalization based on 3'UTR length in male germ cells

Ying Zhang,[1*] Chong Tang,[1*] Tian Yu,[2] Ruirui Zhang,[1] Huili Zheng,[1] Wei Yan[1,2,3]

[1]Department of Physiology and Cell Biology, University of Nevada School of Medicine, 1664 North Virginia Street, MS575, Reno, NV 89557; [2]Department of Biology, University of Nevada, Reno, 1664 North Virginia Street, MS575, Reno, NV 89557, USA

*Equal contribution

This file contains six supplemental figures (Figures S1-S10) and eleven supplemental tables (Tables S1-S11).

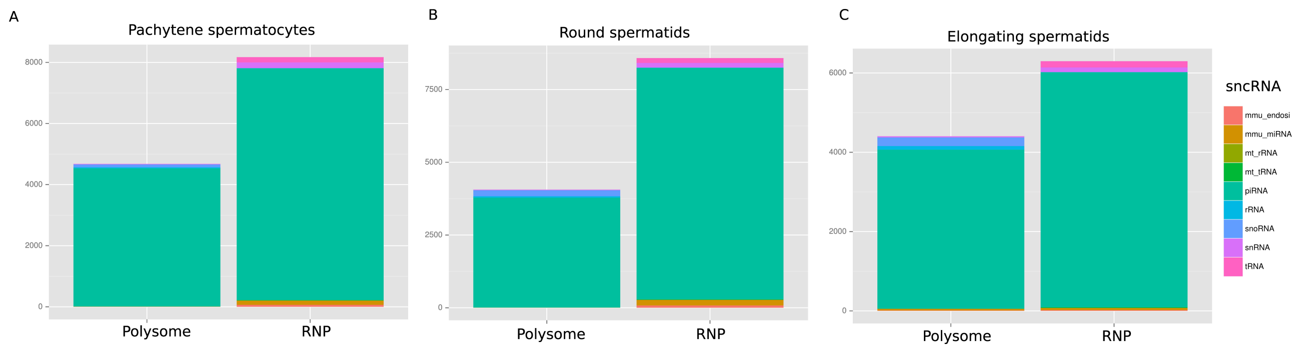

Supplemental Figure S1. Distribution of small noncoding RNAs (sncRNAs) in RNP and polysome fractions in pachytene spermatocytes, round spermatids and elongating spermatids with piRNAs included. sncRNAs preferentially enriched in RNP or polysome fractions in pachytene spermatocytes (**A**), round spermatids (**B**) and elongating spermatids (**C**). RNP-enriched sncRNAs were defined by $\log_2$ (levels in polysome/levels in RNP) < 0 (student's t-test, $p < 0.05$), whereas polysome-enriched sncRNAs were those with $\log_2$ (levels in polysome/levels in RNP) > 0 (student's t-test, $p < 0.05$). The y-axis represents the total number of fraction-enriched sncRNA species.

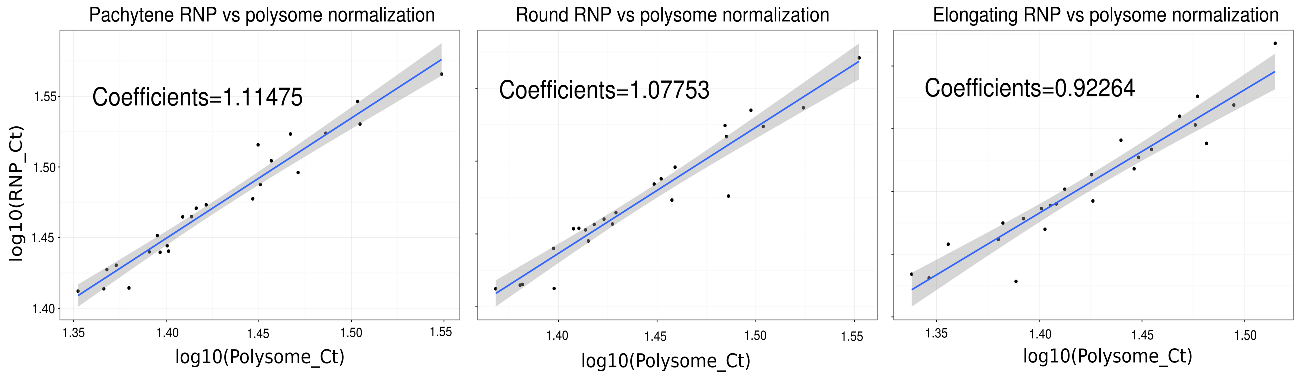

Supplemental Figure S2. Validation of the normalization method used for quantitative comparison of transcript expression levels between RNP and polysome based on RNA-Seq data. For FPKM calculation, the library size factor was set to 1. FPKMs and fragment counts are scaled based on the median of geometric means of fragment counts across all libraries. During fractionation using sucrose gradient, numerous RNAs became free from their binding proteins and got equally distributed across all layers of the gradient. These free RNAs offered natural controls for geometric normalization. To validate the quantification results derived from geometric normalization-based bioinformatic analyses, we randomly chose 24 mRNAs, which displayed equal distributions between RNP and polysome fractions in all three spermatogenic cell types studied [i.e., pachytene (Pachytene) spermatocytes, round (Round) and elongating (Elongating) spermatids], and performed qPCR analyses. The log10(Ct) values of polysome vs. RNP were plotted and the coefficient values were calculated.

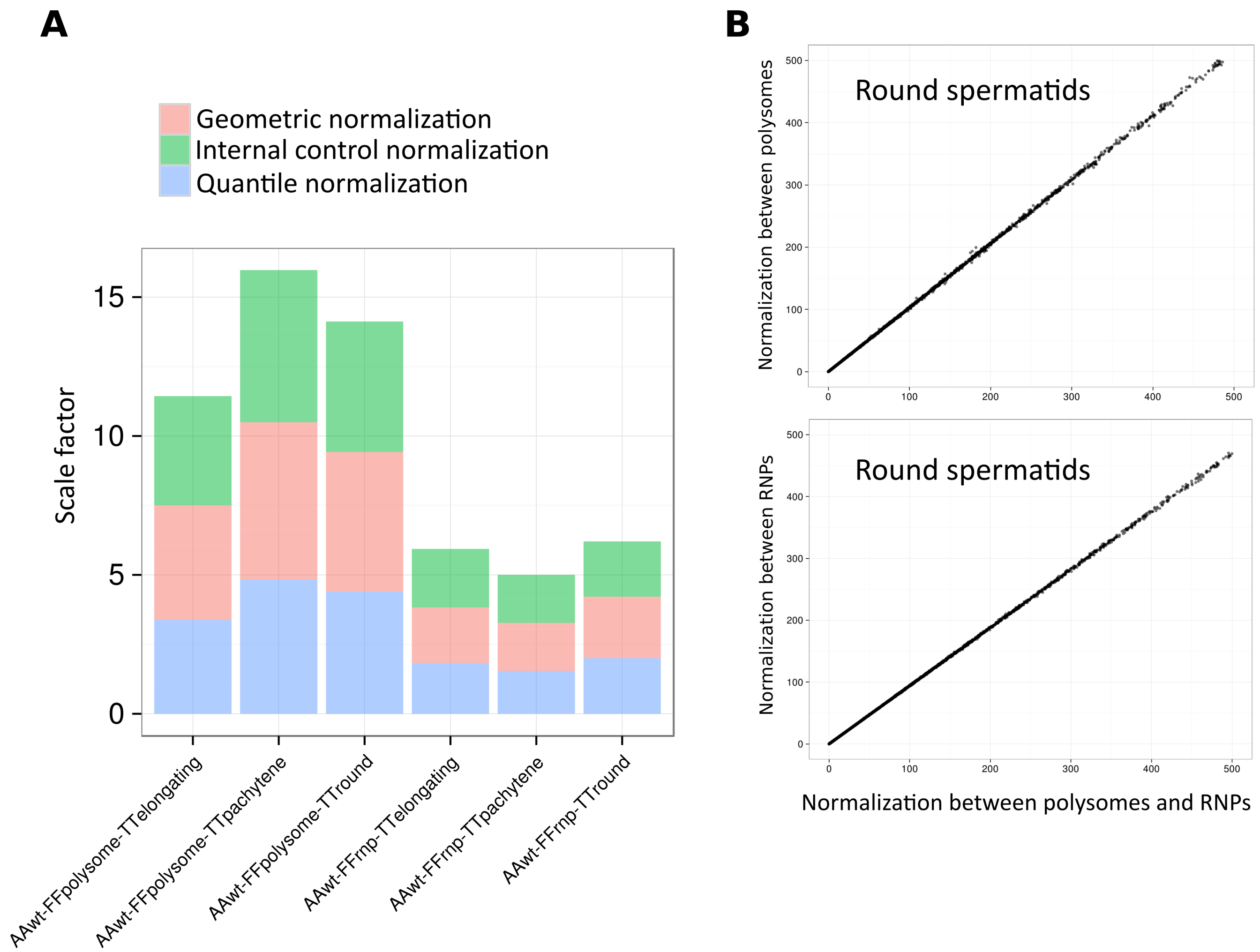

Supplemental Figure S3. Cross validation of the normalization method used in this study. (**A**) Three normalization methods (linear regression normalization using 24 equally distributed mRNAs as internal controls, geometric and quantile normalization) were used to calibrate the same sets of RNA-Seq data from RNP and polysome fractions of pachytene spermatocytes, round and elongating spermatids. The three normalization methods generated similar scale factors, supporting the validity of the normalization method used in this study (i.e., linear regression normalization based on 24 internal control mRNAs) and the robustness of our experimental data. (**B**) Similar FPKMs were obtained from normalization between the same (RNP *vs.* RNP or polysome *vs.* polysome) and different fractions (RNP *vs.* polysome) in round spermatids.

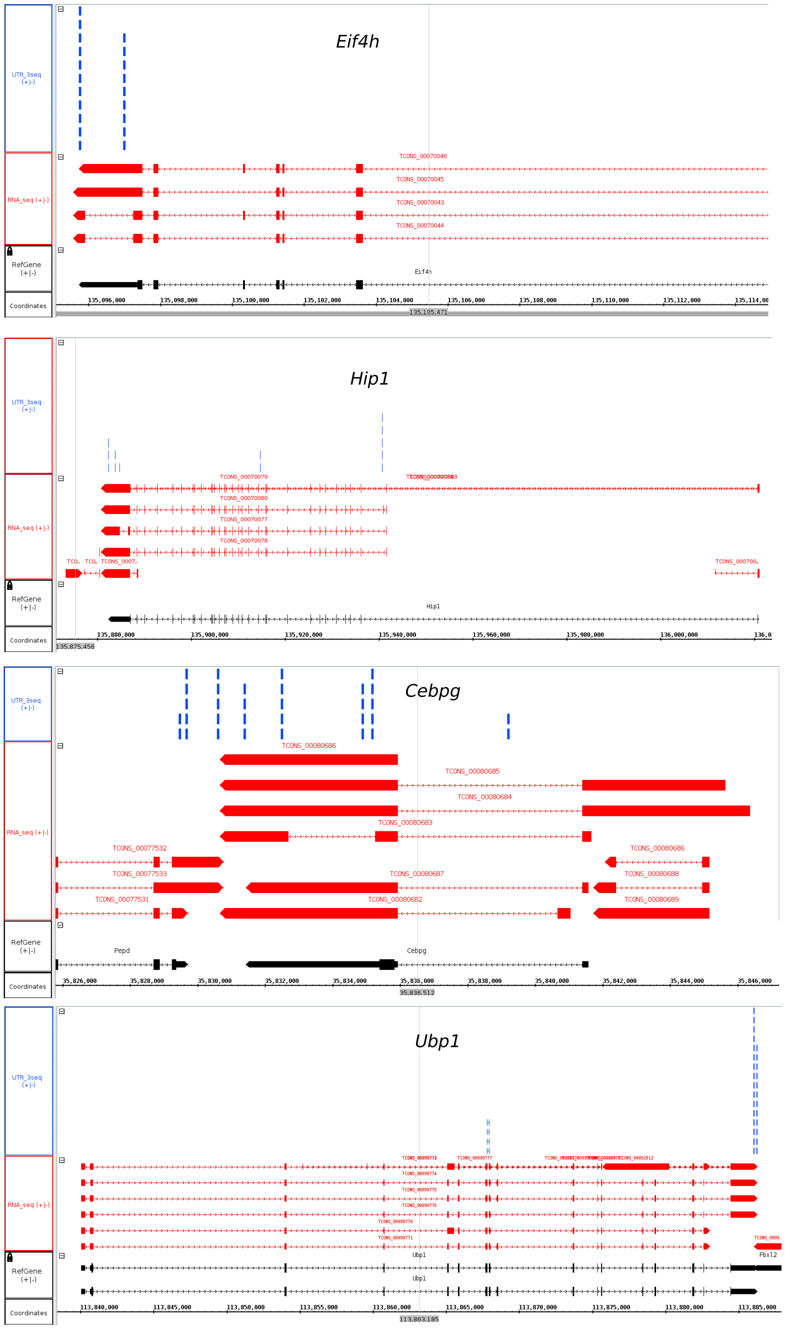

Supplemental Figure S4. Validation of the 3'UTR ends determined by SpliceR using Poly-A-Seq data. After mapping the Polyadenylation Site (PAS)-Seq data of whole mouse testes to the UCSC mm9 genome, the PAS alignment file (blue track) and our Cufflink-assembled file (red track), as well as the genome (black track) were put into the Integrated Genome Browser to visualize the positions of the 3'UTR ends. Results for four representative genes (*Eif4h, Hip1, Cebpg* and *Ubp1*) are shown. The 3'UTR ends of these four mRNAs are consistent with those identified by polyA-Seq.

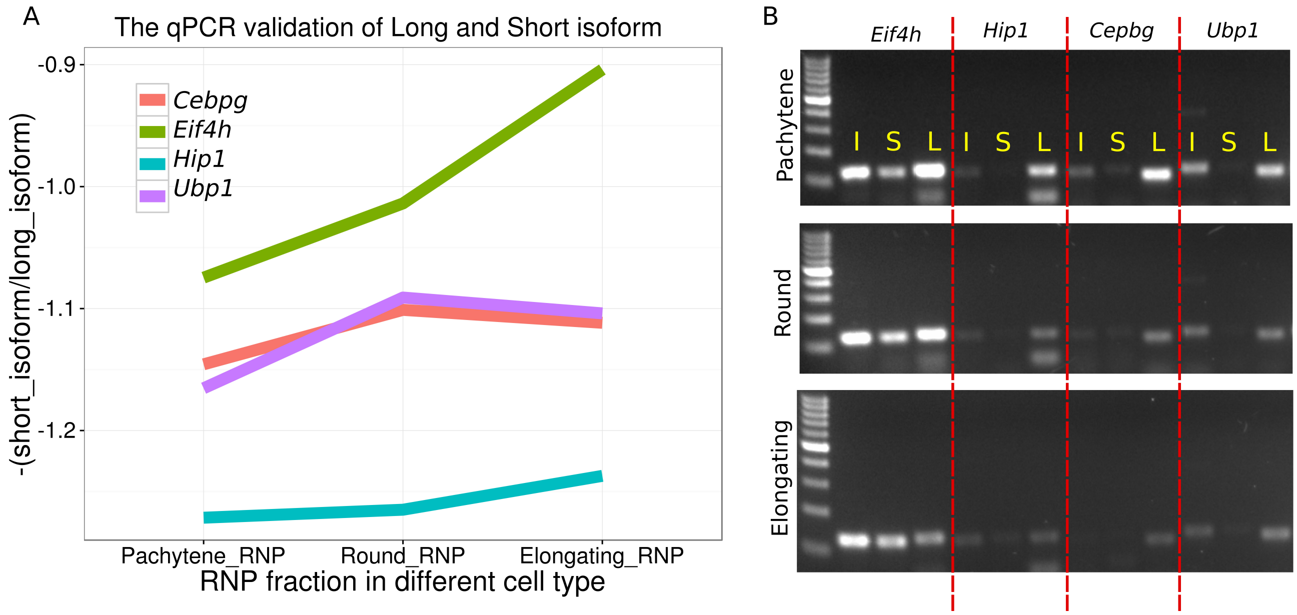

Supplemental Figure S5. Validation of expression levels of the long and short isoforms of four genes (*Cebpg, Eif4h, Hip1* and *Ubp1*) in the RNP fractions of pachytene spermatocytes, round and elongating spermatids. (**A**) qPCR-based validation. The Ct value ratios (Ct of the short isoform/Ct of the long isoform) increased in the RNP fractions from pachytene spermatocytes, to round and then to elongating spermatids. (**B**) Visualization of the relative expression levels of the long and short isoforms of the 4 genes in the RNP fractions in pachytene spermatocytes, round and elongating spermatids using semi-quantitative PCR. "I, S, L" stand for the common region, the short isoform, and the long-isoform, respectively. Note that levels of the long isoforms significantly decreased from pachytene spermatocytes to round and then to elongating spermatids.

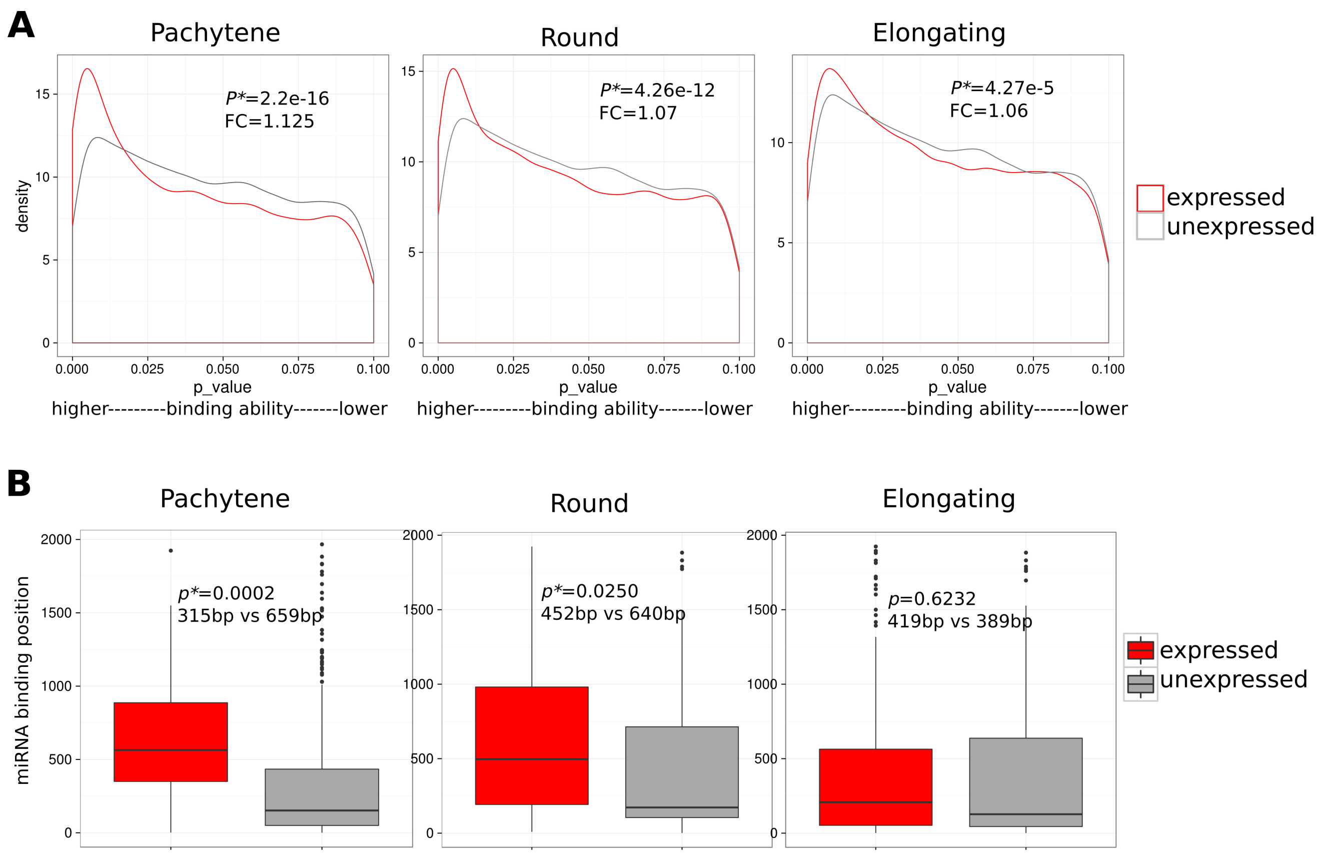

Supplemental Figure S6. Polysome-enriched miRNAs preferentially target the distal sites in the 3'UTRs of polysome-enriched mRNAs. (**A**) Differential binding capacities of polysome-enriched and unexpressed (100 miRNAs without significant expression in the three spermatogenic cells types analyzed) miRNAs to the 3'UTRs of polysome-enriched mRNAs using RNAhybrid. Note that the binding energy of polysome-enriched miRNAs was significantly higher than that of unexpressed miRNAs in all three spermatogenic cell types (pachytene spermatocytes, round and elongating spermatids). FC: average fold change in binding capacity (polysome-enriched/non-polysome-enriched). (**B**) Differential binding positions of polysome-enriched and unexpressed (controls) miRNAs in the 3'UTRs of polysome-enriched mRNAs. Note that the polysome-enriched miRNAs tend to bind more distal sites in the 3'UTRs of polysome-enriched mRNAs compared to those unexpressed miRNAs.

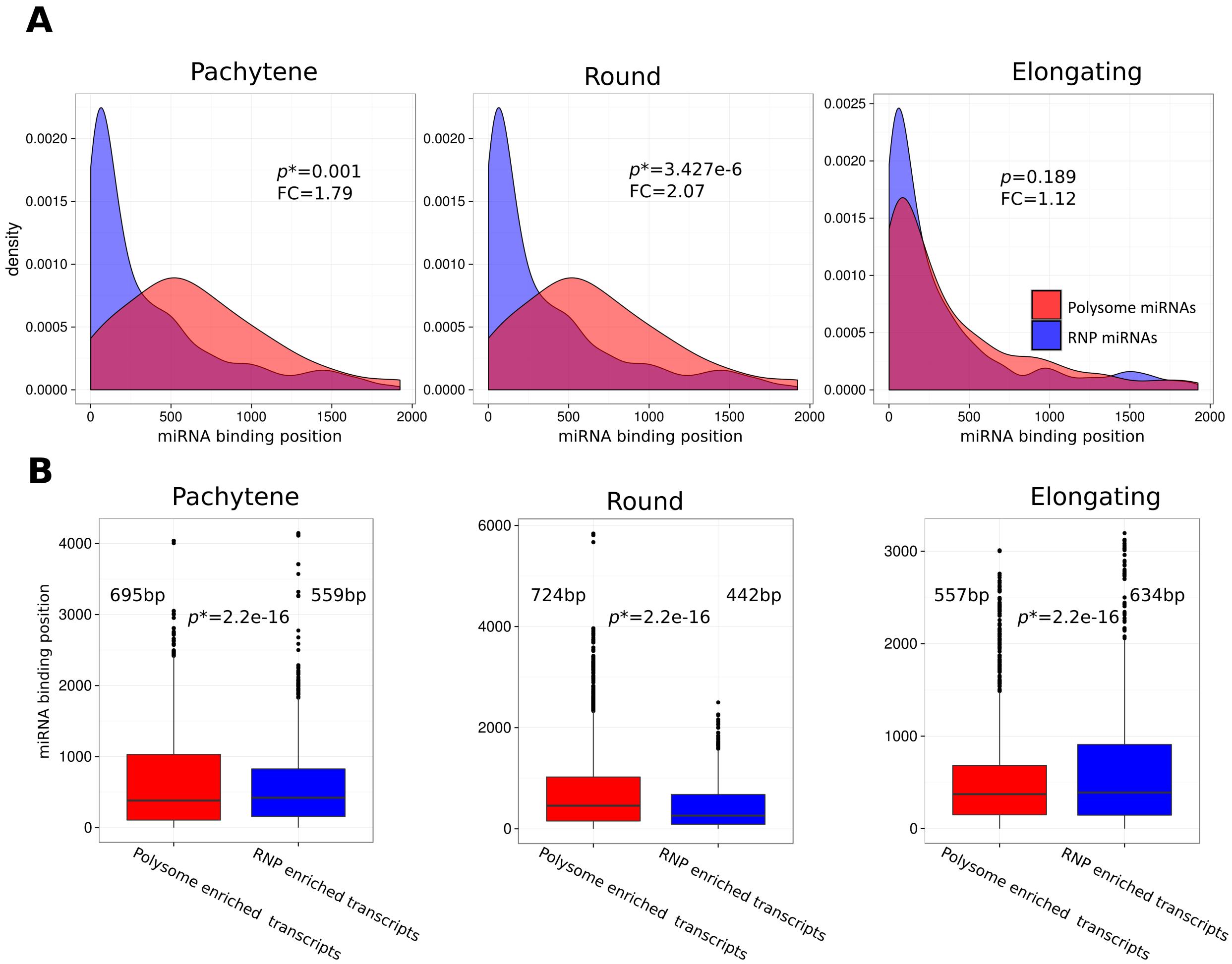

Supplemental Figure S7. Relationship between polysome-/RNP-enriched miRNAs and polysome-/RNP-enriched mRNAs in three spermatogenic cell types, as revealed by miRNA-mRNA binding assays using both RNAhybrid and TargetScan. (A) RNAhybrid-based miRNA-mRNA binding assays showing that the binding sites of RNP-enriched miRNAs tend to be proximal to the stop codon, whereas polysome-enriched miRNAs prefer binding the sites distal to the stop codon in 3'UTRs of mRNAs expressed in three spermatogenic cell types including pachytene spermatocytes, round and elongating spermatids. FC: average fold change of binding positions between polysome- and RNP-enriched miRNAs. **(B)** TargetScan-based miRNA target identification, showing that the targeting sites of miRNAs in RNP-enriched mRNAs are closer to the stop codon than those in polysome-enriched mRNAs in both pachytene spermatocytes and round spermatids. The opposite relationship exists in elongating spermatids, probably reflecting the massive release of the formerly RNP-enriched mRNAs due to the demise of the chromatid body and more efficient translation in the elongation stage of spermatogenesis.

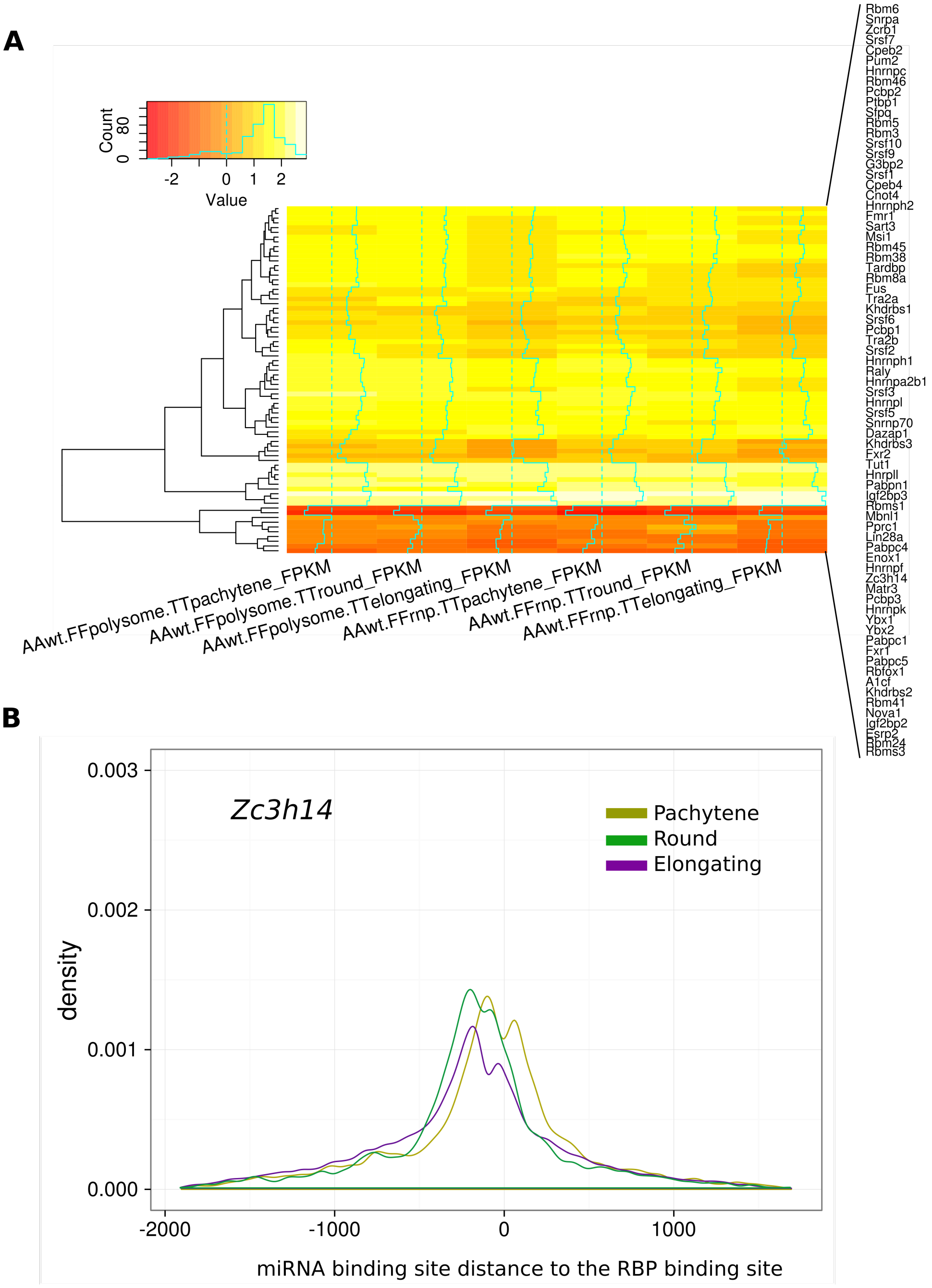

Supplemental Figure S8. Relationship between the binding sites of miRNAs and RNA-binding proteins (RBPs) in 3'UTRs of mRNAs expressed in the three spermatogenic cell types. (**A**) Heat map showing expression of 73 mRNAs encoding RBPs in the RNP and polysome fractions of three spermatogenic cell types. Data were extracted from our RNA-Seq datasets. Note that these RBP mRNAs are generally abundant and display even distribution between RNP and polysome fractions in all three spermatogenic cell types. (**B**) Distribution of RBP- and miRNA-binding sites in the 3'UTR of *Zc3h14* mRNA. RBP-binding sites were determined using RBPmap (http://rbpmap.technion.ac.il/) and miRNA targeting sites were identified using RNAhybrid. Note that the miRNA- and RBP-binding sites are close to each other, suggesting potential interactions between these two types of post-transcriptional regulators.

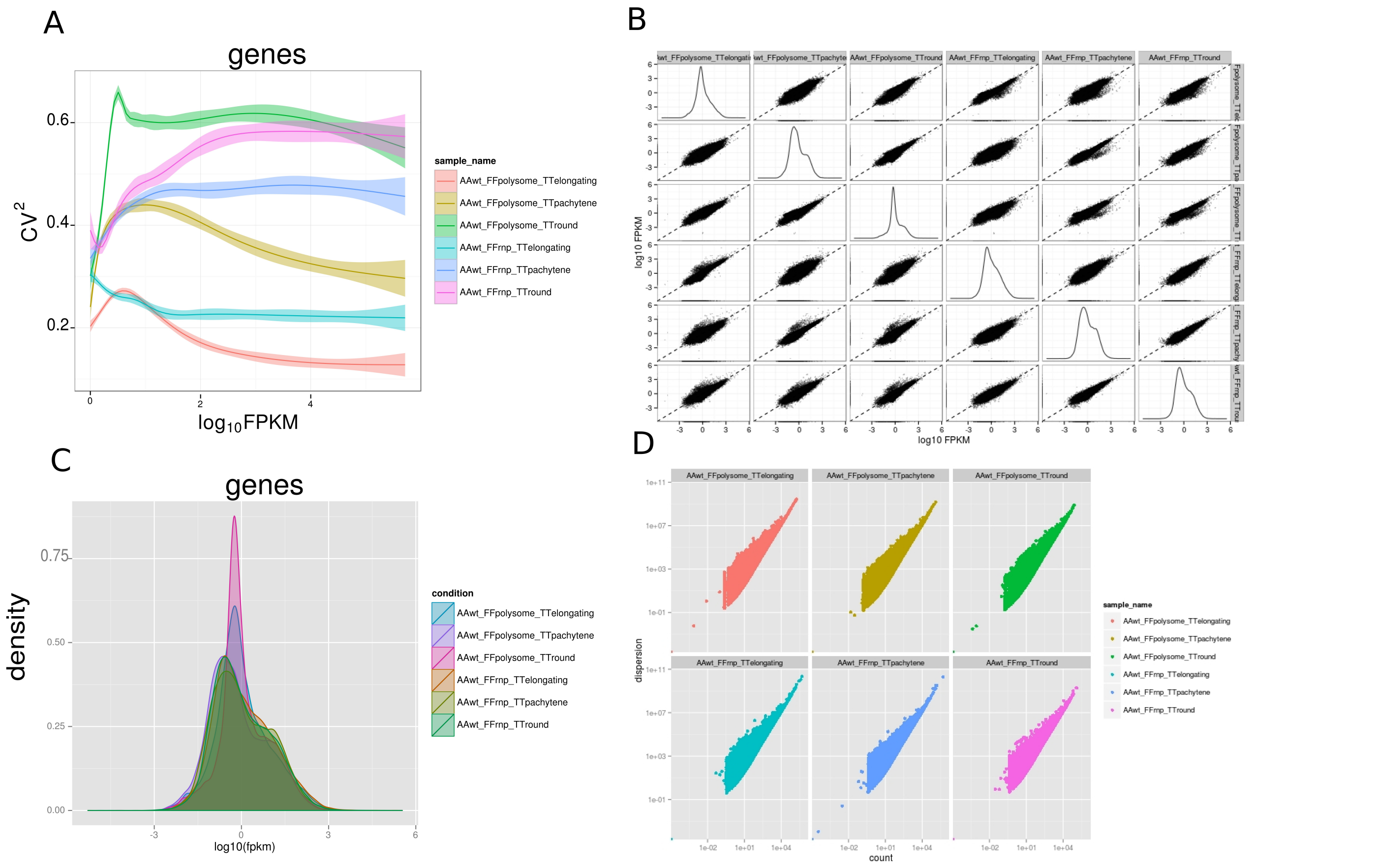

Supplemental Figure S9. Evaluation of the quality of the RNA-Seq data generated in this study. (**A**) Variations among biological replicates of the six RNA samples (pachytene RNP, pachytene polysome, round spermatid RNP, round spermatid polysome, elongating RNP and elongating polysome). The biological variation is reflected by the coefficient of variation to the power of two (CV^2) of FPKM values for each gene. The CV^2 represents a normalized measure of cross-replicate variability, which has been widely used for evaluating quality of RNA-Seq data. The data presented here show that the abundance of the genes varied between replicate RNA samples, especially for the ones with lower FPKM values, which is expected. (**B**) Scatterplot matrix showing the pairwise scatterplots of the $\log_{10}$ normalized FKMP scores across biological replicates of all six RNA samples. (**C**) Density plots showing the distribution of $\log_{10}$ normalized FPKM scores across biological replicates of all six RNA samples. (**D**) Overdispersion plots demonstrating the estimated overdispersion for each sample as a quality control measure.

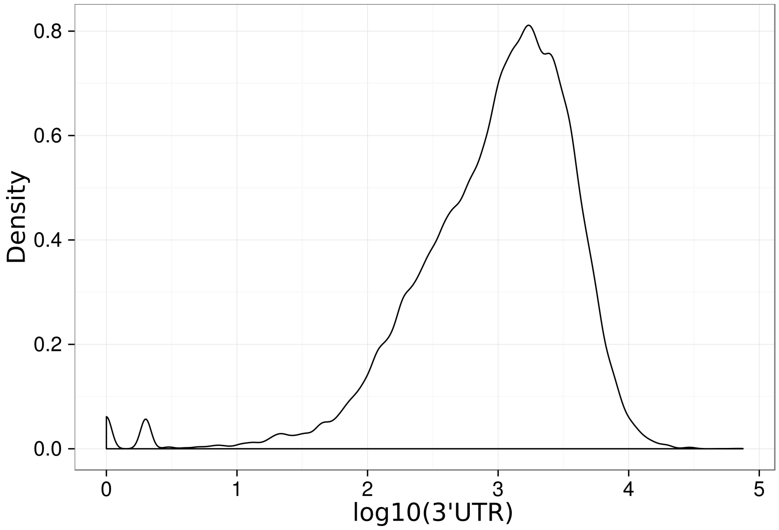

Supplemental Figure S10. Density plot of the log10 (3'UTR length) values of all 69,013 transcripts identified by SpliceR and corrected by the Poly-Seq data showing normal distribution of the data (Median=3).

Table S1.The number of mRNAs preferentially enriched in either RNP or polysome fractions of pachytene spermatocytes, round and elongating spermatids.

	Pachytene spermatocyte	Round spermatid	Elongating spermatid
**RNP-enriched***	679	762	793
**Polysome-enriched***	355	752	422

*The RNP-enriched transcripts are defined by RNP FPKM > polysome FPKM with $p < 0.1$, FPKM> 1, whereas the polysome-enriched transcripts are defined by RNP FPKM < polysome FPKM with $p < 0.1$, FPKM> 1.

Table S2. The average length of mRNAs enriched in either RNPs or polysomes in pachytene spermatocytes, round and elongating spermatids.

	Pachytene spermatocyte	Round spermatid	Elongating spermatid
**RNP-enriched mRNAs**	2,845bp	2,770bp	2,848bp
**Polysome-enriched mRNAs**	3,537bp	4,146bp	3,251bp
**Student's *t*-test, p-value**	4.6e-5	2.2e-16	0.025
**Wilcoxon rank sum test**	2.2e-7	2.2e-16	6.6e-6

Table S3. The average 5'UTR* length of mRNAs enriched in either RNPs or polysomes in pachytene spermatocytes, round and elongating spermatids.

Average 5'UTR length (nt)	Pachytene spermatocyte	Round spermatid	Elongating spermatid
**RNP-enriched**	213	169	276
**Polysome-enriched**	220	276	286
**Student's *t*-test, p-value**	0.87	1.97e-6	0.8845
**Wilcoxon rank sum test**	0.63	2.2e-16	9.737e-11

*The 5'UTR length was determined based on the start site and total length of CDS transcripts identified by SpliceR.

Table S4. The average 3'UTR* length of mRNAs enriched in either RNPs or polysomes in pachytene spermatocytes, round and elongating spermatids.

3'UTR length (nt)	Pachytene spermatocyte	Round spermatid	Elongating spermatid
**RNP-enriched**	1,058	861	798
**Polysome-enriched**	1,351	2,093	1,396
**Student's *t*-test, p-value**	0.0023	2.2e-16	8.73e-11
**Wilcoxon rank sum test, p-value**	2.802e-05	2.2e-16	2.2e-16
**Log10(3'UTR), student's *t*-test, p-value**	7.969e-06	2.2e-16	2.2e-16

*The 3'UTR length was determined based on the stop site and total length of CDS transcripts identified by SpliceR.

Table S5. Chi-Square test for relationship between RNP-enriched miRNAs and their target mRNAs in pachytene spermatocytes.

Pachytene spermatocyte	mRNAs targeted by RNP-enriched miRNAs[2]	mRNAs not targeted by RNP-enriched miRNAs[3]	Average distance between the stop codon and the miRNA targeting site (bp)[4]
**RNP-enriched mRNAs[1]**	506	173	365
**Polysome-enriched mRNAs[1]**	172	183	461
**Chi-square test, p-value**	2.16e-16		
**Student's *t*-test, p-value**			0.001466
**Wilcoxon rank sum test, p-value**			0.002977

[1]RNP- or polysome-enriched mRNAs were defined by $p< 0.1$.
[2]mRNAs targeted by RNP-enriched miRNAs were defined by $p< 0.05$.
[3]mRNAs that were not targeted by RNP-enriched miRNAs were defined by $p> 0.05$.

Table S6. Chi-Square test for relationship between RNP-enriched miRNAs and their target mRNAs in round spermatids.

Round spermatid	mRNAs targeted by RNP-enriched miRNAs[2]	mRNAs not targeted by RNP-enriched miRNAs[3]	Average distance between the stop codon and the miRNA targeting site (bp)
RNP-enriched mRNAs[1]	572	190	311
Polysome-enriched mRNAs[1]	485	267	552
Chi-square test, p-value	2.259e-12		
Student's *t*-test, p-value			2.2e-16
Wilcoxon rank sum test			2.2e-16

[1]RNP or polysome-enriched RNAs were defined by $p< 0.1$.
[2]mRNAs targeted by RNP-enriched miRNAs were defined by $p< 0.05$.
[3]mRNAs not targeted by RNP-enriched miRNAs were defined by $p > 0.05$.

Table S7. Chi-Square test for relationship between RNP-enriched miRNAs and their target mRNAs in elongating spermatids.

Elongating spermatid	mRNAs targeted by RNP-enriched miRNAs[2]	mRNAs not targeted by RNP-enriched miRNAs[3]	Average distance between the stop codon and the miRNA targeting site (bp)
**RNP-enriched RNAs[1]**	347	446	346
**Polysome-enriched RNAs[1]**	145	277	452
**Chi-square test, p-value**	0.001		
**Student's *t*-test, p-value**			0.00213
**Wilcoxon rank sum test, p-value**			0.002433

[1]The RNP- or polysome-enriched RNAs are defined by $p < 0.1$.
[2]mRNAs targeted by RNP-enriched miRNAs were defined by $p< 0.05$.
[3]mRNAs not targeted by RNP-enriched miRNAs were defined by $p> 0.05$.

Table S8. Chi-Square test for relationship between polysome-enriched miRNAs and their target mRNAs in elongating spermatids.

Elongating spermatid	mRNAs targeted by polysome-enriched miRNAs	mRNAs not targeted by polysome-enriched miRNAs
**RNP-enriched mRNAs**	434	359
**Polysome-enriched mRNAs**	157	265
**Chi-square test, p-value**	1.707e-8	

[1]The RNP- or polysome-enriched RNAs are defined by $p < 0.1$.
[2]mRNAs targeted by polysome-enriched miRNAs were defined by $p< 0.05$.
[3]mRNAs not targeted by polysome-enriched miRNAs were defined by $p> 0.05$.

Table S9. Chi-square test for examining the relationship between mRNA movement and miRNAs.

	mRNAs targeted by 216 upregulated miRNAs in RNPs[2]	mRNAs not targeted by 216 upregulated miRNAs in RNPs [3]
**mRNAs significantly upregulated in RNPs from pachytene spermatocytes to round spermatids[1]**	581	328
**mRNAs significantly downregulated in RNPs from pachytene spermatocytes to round spermatids[1]**	837	231
**Chi-square test, p-value**	1.636e-12	

[1]The mRNAs significantly up- or down-regulated from pachytene RNP to round RNP are defined by $p < 0.1$.
[2]mRNAs targeted by upregulated RNP miRNAs were defined by $p< 0.05$.
[3]mRNAs not targeted by upregulated RNP miRNAs were defined by $p> 0.05$.

Table S10. Chi-square test for relationship between mRNA movement and miRNAs.

	mRNAs targeted by 47 shifted miRNAs[2]	mRNAs not targeted by 47 shifted miRNAs[3]
**mRNAs significantly upregulated in polysomes from round spermatids to elongating spermatids[1]**	752	648
**mRNAs significantly downregulated in polysomes from round spermatids to elongating spermatids[1]**	1,301	1,420
**Chi-square test, p-value**	0.00037	

[1]The mRNAs significantly up- or down-regulated from round polysome to elongating are defined by $p< 0.1$.
[2]mRNAs targeted by shifted 47 miRNAs in elongating spermatids were defined by $p< 0.05$.
[3]mRNAs not targeted by shifted 47 miRNAs in elongating spermatids were defined by $p> 0.05$.

Table S11. Sequencing depth and mapping rate of RNA-Seq data

Filename	Sequencing Depth (Reads)	Mapped Reads (Mapping ratio)
AAwt-FFpolysome-TTelongating-SS1	39,631,708	36,746,416 (92.7%)
AAwt-FFpolysome-TTelongating-SS2	42,255,866	39,096,956 (92.5%)
AAwt-FFpolysome-TTelongating-SS3	42,827,937	39,000,891 (91.1%)
AAwt-FFpolysome-TTpachytene-SS1	43,266,421	39,752,391 (91.9%)
AAwt-FFrnp-TTpachytene-SS1	35,836,183	31,907,596 (89.0%)
AAwt-FFrnp-TTpachytene-SS2	34,126,517	28,730,390 (84.2%)
AAwt-FFrnp-TTpachytene-SS3	30,426,572	24,097,602 (79.2%)
AAwt-FFrnp-TTround-SS1	38,876,517	36,032,122 (92.7%)
AAwt-FFrnp-TTround-SS2	38,132,923	32,966,406 (86.5%)
AAwt-FFrnp-TTround-SS3	37,545,584	33,427,628 (89.0%)
AAwt-FFpolysome-TTpachytene-SS2	33,679,781	30,683,632 (91.1%)
AAwt-FFpolysome-TTpachytene-SS3	47,810,323	44,120,866 (92.3%)
AAwt-FFpolysome-TTround-SS1	43,842,908	39,528,128 (90.2%)
AAwt-FFpolysome-TTround-SS2	39,277,819	35,318,906 (89.9%)
AAwt-FFpolysome-TTround-SS3	47,551,095	43,129,626 (90.7%)
AAwt-FFrnp-TTelongating-SS1	38,521,802	35,317,786 (91.7%)
AAwt-FFrnp-TTelongating-SS2	37,872,447	34,273,667 (90.5%)
AAwt-FFrnp-TTelongating-SS3	49,401,156	45,412,738 (91.9%)
